# PolySialic acid-nanoparticles inhibit macrophage mediated inflammation through Siglec agonism: a potential treatment for age related macular degeneration

**DOI:** 10.3389/fimmu.2023.1237016

**Published:** 2023-11-16

**Authors:** Anitha Krishnan, Victor G. Sendra, Diyan Patel, Amit Lad, Michelle K. Greene, Peter Smyth, Samantha A. Gallaher, Úna M. Herron, Christopher J. Scott, Mohamed Genead, Michael Tolentino

**Affiliations:** ^1^ Aviceda Therapeutics Inc., Cambridge, MA, United States; ^2^ The Patrick G Johnston Centre for Cancer Research, School of Medicine, Dentistry & Biomedical Sciences, Queen’s University Belfast, Belfast, United Kingdom; ^3^ Department of Ophthalmology, University of Central Florida School of Medicine, Orlando, FL, United States

**Keywords:** AMD (age-related macular degeneration), polysia mimetics, nanoparticles, macrophages, Siglec

## Abstract

Age-related macular degeneration (AMD) is a chronic, progressive retinal disease characterized by an inflammatory response mediated by activated macrophages and microglia infiltrating the inner layer of the retina. In this study, we demonstrate that inhibition of macrophages through Siglec binding in the AMD eye can generate therapeutically useful effects. We show that Siglecs-7, -9 and -11 are upregulated in AMD associated M0 and M1 macrophages, and that these can be selectively targeted using polysialic acid (PolySia)-nanoparticles (NPs) to control dampen AMD-associated inflammation. *In vitro* studies showed that PolySia-NPs bind to macrophages through human Siglecs-7, -9, -11 as well as murine ortholog Siglec-E. Following treatment with PolySia-NPs, we observed that the PolySia-NPs bound and agonized the macrophage Siglecs resulting in a significant decrease in the secretion of IL-6, IL-1β, TNF-α and VEGF, and an increased secretion of IL-10. *In vivo* intravitreal (IVT) injection of PolySia-NPs was found to be well-tolerated and safe making it effective in preventing thinning of the retinal outer nuclear layer (ONL), inhibiting macrophage infiltration, and restoring electrophysiological retinal function in a model of bright light-induced retinal degeneration. In a clinically validated, laser-induced choroidal neovascularization (CNV) model of exudative AMD, PolySia-NPs reduced the size of neovascular lesions with associated reduction in macrophages. The PolySia-NPs described herein are therefore a promising therapeutic strategy for repolarizing pro-inflammatory macrophages to a more anti-inflammatory, non-angiogenic phenotype, which play a key role in the pathophysiology of non-exudative AMD.

## Introduction

Age-related macular degeneration (AMD) and its complications are the leading cause of blindness and visual loss in adults over 60 years of age, affecting approximately 196 million people in 2020 and likely to affect 288 million by 2040 (1). Clinically, AMD is first detectable with the formation of drusen deposits and pigmentary changes that do not objectively affect vision in the early stages of the condition. With time, patients can develop the divergent vision threatening complication of geographic atrophy (advanced dry) or neovascular (advanced wet) AMD. Drusen deposits are formed between the retinal pigment epithelium (RPE) and Bruch’s membrane, composed of yellow-colored deposits containing proteins, extracellular matrix, and complement components (2). As drusen increase in size and number, AMD progresses with RPE and photoreceptor degeneration and the pathological growth and leakage of the choroidal vasculature leading to exudation, hemorrhage, fibrosis and subsequent vision loss (3). Macrophages and microglia migrate towards the Bruch’s membrane and begin to express INOS a sign of NF-kappaB activation and cellular polarization (4).

Transcriptomic analyses of healthy and AMD human eyes revealed that chemokine, cytokine and complement cascade signaling pathway genes are upregulated, suggesting that the recruitment and activation of immune cells such as macrophages/microglia as well as complement occurs during AMD development (5, 6). Retinal microglia are the resident inflammatory cells of the retina and share similarities with tissue macrophages and central nervous system microglia. They reside in the inner layers of the neural retina near retinal blood vessels, and their numbers increase with age (7). Aged microglia have a resting phenotype with a branched morphology and show decreased responsiveness to tissue injury (7). In AMD, microglia accumulation in the subretinal space is a response to both inflammation and an attempt to promote healing. However, the exacerbation of this infiltration and accumulation of microglia and macrophages, particularly M1 phenotype, can promote both neovascular lesion growth and, retinal degeneration leading to geographic atrophy (8–10). Pro-angiogenic factors such as vascular endothelial growth factor A (VEGFA) is produced by recruited macrophages and RPE cells in the retina (10–13). In addition, the presence of proinflammatory mediators such as TNF-α, IFN-γ, and IL-1β can activate macrophages and microglia in the mouse retina, promoting dysfunction and death of RPE cells, induce photoreceptor/RPE cell apoptosis, increase VEGF expression, and causing the breakdown of the blood-retinal barrier (14, 15). The main treatment for exudative form of AMD (16) targets pathological neovascularization by using anti-VEGF antagonists but has been shown to be effective in only a subset of patients (17, 18). There are several candidate agents under study with some in a late clinical trial phase based largely on modulating the complement cascade and/or other mechanisms that lead to cell degeneration (19–21). Recently a complement C3 inhibitor and a C5 inhibitor have been FDA approved for the treatment of geographic atrophy but have not demonstrated improvement in visual function, have produced some serious adverse events and barely reduce the growth rate of the geographic atrophy (22).

Sialic acids are widely expressed as terminal sugars on glycans of glycoproteins and glycolipids in eukaryotic plasma membranes and are central to immune surveillance and homeostasis by displaying self-associated molecular patterns (23). Sialic acids can be recognized specifically by a family of receptors called Siglecs (sialic-acid-binding immunoglobulin-type lectins) expressed on the surfaces of subsets of immune cells CD33-related Siglecs (CD33rSiglecs). Upon engagement by sialic acid (Sia), various members of the Siglec family act as immune checkpoints involved in a range of immune responses providing opportunities to target Siglecs therapeutically (24–27). Siglecs-7 and -9, as well as their mouse ortholog Siglec-E, are expressed on macrophages, microglia and neutrophils, while Siglec-11 is found exclusively on microglial cells and play key roles as immune checkpoints or resolution receptors with the ability to attenuate cellular pro-inflammatory responses (28–33). These Siglecs are involved in maintaining immune homeostasis and preventing excessive inflammation.

Siglecs mediate their inhibitory effects on toll like receptor (TLR)-induced cytokine production in macrophages/microglia via recruitment of the serine/threonine phosphatase tyrosine specific Src homology-2 domain-containing phosphatase (SHP)-1 and SHP-2 to the cytosolic ITIM domain which result in potent deactivation of proinflammatory signaling in the cell (29, 34–36). Because of the potential to globally deactivate inflammatory cells, Sialic acid mimetic Siglec agonists could provide an effective tool to determine the importance of Siglec signaling in the development of both dry and wet AMD.

Siglecs have previously attracted attention as potential therapeutic targets for treating autoimmune diseases, sepsis, acute respiratory distress syndrome and other inflammatory conditions (37–41). Previously, it was demonstrated that a di-Sia-functionalized NP was more effective in reducing macrophage-induced inflammatory response in a Siglec-E dependent manner compared to the free unconjugated disialic acid ligand, demonstrating elegantly the potency of sialic acid avidity and receptor crosslinking provided by NP multi valent display of sialic acid ligands (42, 43). In this report we describe the therapeutic utility of presenting more complex sialic acid pattern (polysialic acid) on a nanoparticle platform to agonize Siglec receptors expressed in macrophages associated with AMD. First, we determined the expression of Siglecs -7, -9 and -11 in retina tissues from AMD patients and found that they are markedly upregulated compared to normal controls. Interestingly, we found correlation of Siglec expression with M0 and M1 macrophages in the AMD patients. We then show that the PolySia-NPs can bind Siglec receptors on macrophages and elicit anti-inflammatory effects *in vitro* and *in vivo* murine models of AMD. Thus, we propose that a novel PolySia-NPs platform is a safe and effective immunomodulator of macrophage functions via binding to Siglecs, with the potential for therapeutic application in AMD. Furthermore, the demonstrated *in vivo* safety and efficacy of intravitreal injections of PolySia-NP to treat animal models of AMD enabled an FDA IND filing and approval to enter into a human Phase II/III Clinical trial for the treatment of macular degeneration (geographic atrophy).

## Methods

### Transcriptomic analysis of AMD eyes

Transcriptomic analysis was performed to investigate the expression changes of Siglecs in AMD eyes. The GSE135092 dataset, which included RNA-seq data from eyes with a clinical diagnosis of AMD using the AREDS classification and ages ranging from 59 to 98 years, was utilized for this analysis (44). The dataset consisted of bulk RNA-seq data from the retina tissues of the macula and non-macula (peripheral) regions of 129 postmortem donors (106 control and 23 AMD patients). The analysis was conducted using R software (45). Statistical analysis was performed using the Student’s T-test or the Wilcoxon test in the case of non-parametric data.

The correlation between expression of Siglec-7, -9 and -11 and M0, M1 and M2 macrophages was analyzed by determining the correlation coefficient, R value. In this case, Spearman’s correlation coefficient was used. The M0, M1, and M2 macrophage signatures are derived from xCell (5). Coefficient values range from +1 to -1, where +1 indicates a perfect positive relationship, and -1 indicates a perfect negative relationship. The closer the correlation value is to zero the weaker it is. The p-value indicates the degree of significance (ie. how real the probability is of the two variables being correlated). P-values ≤ 0.05 suggest that the associations (whether strong or weak) occur by chance, and it should be accepted that there is no correlation. P values can be influenced by sample size hence why it’s possible to get weak correlations with highly significant p-values in analyses with large sample sizes. In the data analyzed in macula AMD groups had < 30 samples while control groups had > 100 samples. The following R cutoffs are accepted to verbally describe the correlations (both positive and negative relationships): Very weak = 0-0.19, Weak = 0.2-0.39, Moderate = 0.4-0.59, Strong = 0.6-0.79 and Very strong = 0.8-1.

### Cell lines

THP-1 cell line (ATCC TIB-202™, Gaithersburg, MD). Primary human peripheral blood mononuclear cells (PBMC; Stem Cell Research, Cat No-70500.2).

### Primary macrophages

M1 and M2 Macrophages derived from monocytes (MDM) purified from PBMC. Primary human macrophages were obtained from fresh ¼ pack Leukopak^®^ (Stemcell Technologies, Cambridge, MA). Monocytes were isolated and enriched using CD14 + magnetic beads from EasySep Human Monocyte Isolation kit according to manufacturer’s protocol (Stemcell Technologies; Cambridge, MA). Monocytes purity and Siglec-7, -9 and -11 expression was assessed by flow cytometry by staining with FITC conjugated anti-CD14 and BV421 conjugated anti-CD45 (HCD14 and 2D1, Biolegend, San Diego, CA) as leucocyte markers (**Supplementary Figure 4A**); and APC conjugated anti-Siglec-7, FITC conjugated anti-Siglec-9 (clones S7.7, K8 Biolegend, San Diego, CA), AF647 conjugated anti-Siglec-11 (clone 705904, R&D system, Minneapolis, MN) and the isotypes controls antibodies APC-Mouse IgG1κ, FITC-Mouse IgG1κ (Biolegend, San Diego, CA), Alexa Fluor^®^ 647-Mouse IgG2A (R&D systems) respectively.

The CD14 + enriched cells were frozen and stored in liquid nitrogen until use. For the experiments, approximately 6 days prior to use, the cryopreserved monocytes were thawed and cultured in 24-well plates at 250,000 cells/500 μL with serum-free ImmunoCult™-SF Macrophage Differentiation Medium with 50 ng/mL macrophage colony stimulating factor (MCSF). The medium was used to differentiate the monocytes into M1 macrophages (classically activated) and M2a macrophages (alternatively activated). M1 cells were activated by the addition of LPS (10 ng/mL) (Sigma Aldrich, St. Louis, MO) and interferon-gamma (IFN-γ; 50 ng/mL), while M2 cells were activated by the addition of IL-4 (10 ng/mL; Stemcell Technologies, Cambridge, MA). Alternatively, M1 macrophages were stimulated with Low Density Lipoprotein from Human Plasma, oxidized (OxLDL) (ThermoFisher, Waltham, MA) 10μg/mL instead of LPS. M0 cells were obtained from media without the addition of any activating agents.

### Siglec-Fc binding assay

Recombinant Fc-Siglec-E, -3, -5, -7, -9, -10 and -11 (R&D system, Minneapolis, MN) were coated in a 96 well plate overnight at 4°C according to manufacturer’s protocol (R&D system, Minneapolis, MN). PolySia-NPs and a blank control nanoparticle (NP) were added on coated plates at different concentrations (5−0.1 mg/mL). Blank-NPs are control nanoparticles without PolySia ligand decoration. After 2h incubation, the presence of PEG on the NPs was detected using anti-PEG biotin/horseradish peroxidase (HRP) according to manufacturer’s ELISA protocol. After adding the HRP substrate, the color developed was quantified by measuring the absorbance at 450 nm using Spectra iMax plate reader.

### Competitive binding assay

Panc-1 cells were obtained from American Type Culture Collection (ATCC) and maintained in Dulbeco’s Modified Eagle’s Medium (DMEM) (high glucose) supplemented with 10% foetal bovine serum (FBS), 1% penicillin/streptomycin and 1 mM sodium pyruvate at 37°C with 5% CO2 during continuous culture. 0.25 μg of rhSiglec-7 Fc (1138-SL, R&D), rhSiglec-9 Fc (1139-SL, R&D) or rhIgG1 Fc (110-HG, R&D) was precomplexed with 0.5 μg PE-labelled Goat anti-Human IgG Fc (12-4998-82, Invitrogen) in 0.5% bovine serum albumin (BSA) in PBS for 15 mins at 4°C in the dark before the addition of the nanoparticles. PolySia-NPs or Blank-NP were incubated with the above for 15 min at 4°C, followed by the addition of sialic acid expressing Panc-1 cells (46, 47) for a further 30 mins at 4°C. PE fluorescence was analyzed a via flow cytometry (Accuri C6 Plus, BD Bioscience). Binding of PolySia-NPs to the PE-labelled Siglec Fc proteins would competitively block the binding of the latter to sialic acid expressed on the Panc-1 cell surface, resulting in a reduction in PE fluorescence.

### ELISA assays

The supernatant from the macrophages culture were collected and assessed for cytokines or VEGF by ELISA kits (R&D systems, Minneapolis, MN) according to manufacturer’s instructions. Briefly, clear polystyrene microplates (Cat#DY990, R&D systems, Minneapolis, MN) were coated with capture antibodies overnight. Next day, wells were washed and blocked with reagent diluent for 1h. Repeat the aspiration/wash and add the standards and the cell culture supernatant diluted in reagent diluent. Incubate for 2h at room temperature. After 3x aspiration/wash, incubated with detection antibody for 1h. Aspirate and wash 3X and incubate with Streptavidin-HRP for 20 min. After 3x aspiration/wash, add substrate solution to each well and incubate for 20 min. Add stop solution to each well and determine the optical density of each well by using a microplate reader set to 450 nm.

### MTT-cell viability assays

LPS or OxLDL-activated M1 macrophages derived from PBMC or THP-1 cell line were cultured in presence of the PolySia-NPs and controls at different doses from 0.01-5 mg/mL for THP-1 and 0.01-1 mg/mL for M1 macrophages in a 96-well plate for 24h. Then, cells were washed and incubated with 1 mg/mL of MTT (3-(4,5-dimethylthiazol-2-yl)-2,5-diphenyltetrazolium bromide) from the *in vitro* toxicology assay kit (Sigma Aldrich, St. Louis, MO) for 3-4 hours at 37°C. The viable cells contain NAD(P)H-dependent oxidoreductase enzymes which reduce the MTT to formazan. The resulting formazan crystals were dissolved in 100uL of MTT solubilization buffer and the absorbance was measured at 570nm using Spectra iMax plate reader. Each plate contained a positive control (dimethyl sulfoxide, DMSO), a negative control (untreated cells) and blank (no cells). The values were compared to the control cells. Graphs represent cell viability when compared to untreated control cells.

### *In vivo* safety assessment on mice

Ocular tolerability was evaluated by injecting 2 µL via IVT of PolySia-NPs, PolySia alone, or blank-NPs in 15–16-week-old C57BL/9JRj mice (N=4/group). Baseline spectral-domain optical coherence tomography (OCT) and flash electroretinogram (fERG) measurements were made on day -5. After OCT, a detailed fundoscopic ophthalmic exam was performed. IOP was measured in awake animals on day -2. Animals were subsequently randomized into treatment groups based on fERG a-wave amplitudes. Mice received a single bilateral intravitreal injection (2 µl) of either: PolySia-NPs, Blank-NPs or PolySia only. Ocular tolerability was assessed at 4h, 24h, 48h, 72h, 1wk, 2wk by macroscopic ophthalmic exam and IOP measurements. On Day 14, fERG, OCT, and detailed fundoscopic ophthalmic exam was performed. Ocular tissues were collected and processed as follows: OS – retinal and choroidal flat mounts stained for Iba1 and F4/80. IOP was measured from awake mice using an Icare^®^ TonoLab tonometer (Icare Finland, Finland).

### *In vivo* safety assessment on Dutch Belted rabbits

For toxicity studies in Dutch Belted rabbits, PolySia-NPs were tested at low, medium and high dose by intravitreal (IVT) injection (N=3/group). A group that received a 10% sucrose vehicle was used as control. Ocular examinations (OEs) and tonometry were performed at baseline and on Days 1, 3, 7, and 14. The modified Hackett and McDonald ocular grading system was used to grade inflammation. Optical coherence tomography (OCT) and electroretinography (ERG) were performed at baseline and on Day 14 for PolySia-NPs tested at low, medium and high dose/eye. Body weights were taken at baseline and at the time of necropsy.

### *In vivo* bright light damage mouse model

The bright light damage (BLD) model causes damage to photoreceptors by upregulation of reactive oxygen species (ROS). Retinal damage was induced by 4h exposure to bright light (10,000 lux intensity) in BALB/cAnNCrl mice aged 8 weeks, after which the mice were returned to normal light/dark cycle (10 lux. 12h/12h). The intravitreal (IVT) injection by using a 5 µl glass microsyringe (Hamilton Bonaduz AG, Bonaduz, Switzerland) of:1- PolySia-NPs, 2-Blank-NPs or 3- PolySia Ligand (n=10-11/group) was administered unilaterally into the right eye (OD) one day prior to the induction. All treatments were given as 2μL intravitreal injection administered unilaterally to the right eye (OD) one day prior to BLD. The mice were examined using high-resolution spectral domain optical coherence tomography (SD-OCT) six days prior to the BLD induction and at day seven post-BLD. The mice were sacrificed at day seven after BLD, eyes were enucleated and cryopreserved for histological/immunohistochemical analysis, H&E for retinal thickness and F4/80 immunostaining. 

Another study was performed in humanized Siglec-11 tg mice enrolled into 3 groups (N=5/group). The humanized Siglec-11 transgenic (tg) mice were generated by human SIGLEC-11 (NCBI Reference Sequence: NM_052884.3) knock in (KI) at the locus of ROSA26 gene (NCBI Reference Sequence: NR_027008.1) in C57BL/6N mice by CRISPR/Cas-mediated genome engineering (Cyagen, Santa Clara, CA). For the KI model, the “CAG promoter-Kozak-human SIGLEC11 CDS-rBG pA” cassette were cloned into intron 1 of ROSA26 in reverse orientation (**Supplementary Figure 7A**). To engineer the targeting vector, homology arms were generated by PCR using BAC clone as template. Cas9 and gRNA were co-injected into fertilized eggs with targeting vector for mice production. The gene was introduced into the whole genome, therefore expressed in the whole body. The expression of human Siglec-11 and Siglec-E was demonstrated by genotypification compared to B6 wt mice (**Supplementary Figure 7B**).

On Day -1, animals received a 1 µL intravitreal (IVT) injection into both eyes (OU): 10% sucrose vehicle, low and high dose/eye of PolySia-NPs. On Day 0, retinal degeneration was induced in all animals with exposure to light at 10,000 lux for 4 hours. Ocular examinations (OEs) were performed at baseline and on Day 6. Electroretinography (ERG) and optical coherence tomography (OCT) were performed at baseline and on Day 7. On Day 7, the animals were euthanized; all left eyes (OSs) were processed for Sponsor-performed analyses, N=3 right eyes (ODs) per group were processed for immunohistochemistry (IHC) described below, and N=2 ODs per group were processed for hematoxylin & eosin (H&E) histopathology.

### Spectral Domain Optical Coherence Tomography (SD-OCT)

Retinal structure was assessed and quantified by performing SD-OCT bilaterally at baseline and 7 days after BLD by using the Envisu R2200 SD-OCT system (Bioptigen Inc./Leica Microsystems, USA), where a retinal scan was performed. The scanned area covers a 1.4 x 1.4 mm 2 of the retina centered around the optic nerve. Each scan was composed of 100 B Scans each one composed of 1000 A Scans. The registered and averaged scans were loaded onto the Diver software (v 3.0.8. Bioptigen) for analysis. A 25-point grid was placed centered on the optic nerve head and the thickness of the Outer Nuclear Layer was measured. Furthermore the 25-points grid was divided into quadrants to obtain the thickness on the Superior-Nasal, Inferior-Nasal, Superior-Temporal and Inferior-Nasal regions.

### Ocular examination

A veterinary ophthalmologist performed complete OEs using a slit lamp biomicroscope and indirect ophthalmoscope to evaluate ocular surface morphology, anterior segment, and posterior inflammation on all animals prior to injections to serve as a baseline for enrollment into the study and on Day 6. Pupils were dilated for ocular examination using topical 1% tropicamide HCl (one drop in each eye 15 minutes prior to examination. The modified Hackett and McDonald ocular grading system (48) was used for scoring. Animals were not tranquilized for the examinations.

### Electroretinography

ERG, or electroretinography, is a method for evaluating the functional impairment of the eye by measuring the electrical responses of various nerve cells in the retina. The b-wave amplitude of the ERG reflects the function of the deep retinal cells, where a decrease in the amplitude of the b-wave indicates retinal dysfunction, whereas an increase indicates improvement. ERGs were performed at baseline and on Day 7. Animals were dark-adapted for a period of at least 12 hours prior to ERG. Animals were sedated for ERG utilizing a ketamine/xylazine cocktail (80-90/10-20 mg/kg) administered intraperitoneally (IP). Under dark adaptation, eyes were dilated using a cocktail of tropicamide HCl 1% and phenylephrine HCl 2.5%. Before ERGs were recorded, pupil dilatation was checked to ensure full dilation. Animals were positioned on the ERG machine and proparacaine and GenTeal (Alcon, Switzerland) were applied to the eyes, followed by the electrode contact and reference leads. Animals were placed on a warm water blanket to control body temperature and contact lens leads were placed on the eyes. A reference lead was placed in the mouth and a ground lead was placed near the tail of the animal. ERGs were recorded using scotopic and photopic methods as previously described by A.L Georgiou et al. (49) and as described by ISCEV ERG standards (50).

### *In vivo* laser-induced choroidal neovascularization mouse model

The mouse CNV model was used to recruit the growth of subretinal blood vessels from the choroid by perforating Bruch’s membrane using a diode laser. The contralateral eye remained naïve and served as a control. Blank-NP, PolySia-NPs, PolySia Ligand and Eylea^®^ were injected via intravitreal (IVT) immediately after CNV induction. Aflibercept (Eylea^®^, Bayer Pharma AG, Germany) (N=6/group). Mice were monitored by *in vivo* imaging using fluorescein angiography (FA) on days seven post CNV induction. Following a 48h washout period (day 9), mice were sacrificed by transcardial perfusion. The eyes were post-fixed and choroidal flat mounts were prepared from treated eyes. Choroidal flat mounts were labeled with Isolectin B4 or Iba-1 to quantify the area of neovascularization or macrophage/microglia at Day 2 and 9 post-CNV.

The eyes of humanized Siglec-11 tg mice were treated with a single dose of 1μL IVT injections OU on Day 1 as follow: 1 group received 10% sucrose vehicle (control), 1 group received 0.15 mg/eye PolySia-NPs (3.0mg/mL), and 1 group received 1.0 mg/eye PolySia-NPs (19.19mg/mL) (N=7/group). Afterward, a 532 nm diode laser was used to create 4 single laser spots OU surrounding the optic nerve. Ocular examinations (OEs) were performed at baseline to determine enrollment into the study as described above. Fluorescein angiography (FA) was performed on all animals OU on Day 8 by retinal photography performed approximately 1 minute after IP sodium fluorescein injection (12 mg/kg). Following euthanasia on Day 8, 9 to 10 eyes/group were enucleated, and retinas were dissected for flat mount immunohistochemistry (IHC) as described below.

### Fluorescein angiography

Vascular leakage at the choroid level was examined using a Heidelberg Spectralis HRA system (Heidelberg Engineering, Germany). Briefly, a drop of 0.5% tropicamide (Oftan Tropicamid. Santen Oy) was administered onto the cornea of the anesthetized mouse to dilate the pupils. The mouse was then placed and secured onto a holder for *in vivo* imaging. After aligning the optic nerve head at the retinal level, with the use of the infrared reflectance camera, a solution of 5% sodium fluorescein (Sigma-Aldrich, Germany) was administered as a s.c. injection (30 µL/10 g). Consecutive fluorescent images were taken every 60 sec from the retinal and choroidal focus levels for a period of 5 min after the fluorescein administration.

### Flat mount immunohistochemistry

For IHC, eyes were enucleated and immediately fixed in 4% paraformaldehyde in phosphate-buffered saline (PBS) and stored overnight at 4°C. The following day, the eyes were transferred to cold immunocytochemistry (ICC) buffer (PBS containing 0.5% bovine serum albumin [BSA] and 0.2% Tween 20) until processing. Using a dissecting microscope, the eye was trimmed of extraneous tissue and the anterior segment and lens were removed. The retina was detached using fine curved scissors and discarded. Eye cups were rinsed with cold ICC buffer. Five (5) eye cups/group were incubated in antibody cocktail with 1/100 Isolectin-B4 antibody DyLight 649 (Vector Labs cat# DL-1208-.5) 4h at 4°C and the sclera-choroid/retinal pigment epithelium (RPE) complexes were flat mounted, covered, and sealed. Two-dimensional (2D) fluorescent microscopy images were acquired, digitized, and analyzed using an Olympus Bx63 upright fluorescent microscope and CellSens (Olympus) software and post-acquisition analysis of Isolectin area was performed with CellSens software. To quantify neoformed vessels, the Isolectin-B4 area was measured in µm2.

## Results

### Siglecs -7, -9, and -11 expression is increased in AMD human retinas and macrophages

**Figure 1:**
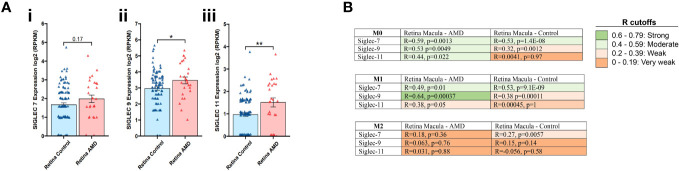
Transcriptomic analysis for Siglec-7, -9 and -11 in retina tissues associated to AMD. Expression of Siglec -7, -9 and -11 was determined on the macular region of the retina from AMD patients compared to control **(Ai-iii)**. Data analysis was performed based on data from publicly available (GSE135092) RNA-seq datasets. Correlation coefficient, R value determined between the expression of Siglec-7, -9 and -11 in M0, M1 and M2 macrophages from macula retinas from AMD patients and controls. R cutoff determines strong (green), moderate (faint green), weak (faint orange) and very weak (orange) correlation. Spearman’s correlation coefficient was used. The M0, M1, and M2 macrophage signatures are derived from xCell platform **(B)**.

In the first stage of our investigations, we examined the expression of the inflammation resolution (ITIM containing) Siglec receptors from human retina donors with AMD (44). Transcriptomic analysis showed significant increases in the expression of Siglec-9 (3.47 vs 2.96, p=0.04) (**Figure 1A**ii) and Siglec-11 (1.51 vs 0.96, p=0.02) (**Figure 1A**iii) in the macular region of AMD retinas compared to healthy controls, while a trend of upregulation (not reaching significance) was observed for Siglec-7 (1.67 vs 1.98, p=0.17; **Figure 1A**i). We also analyzed expression alterations in AMD of Siglec-1, -2, -3, -4, -5, -6 -8, -10, -12, -14 -15, and -16 (**Supplementary Figure 1**).

Next, we assessed the expression of Siglecs 7, 9 and 11 in M0, M1 and M2 macrophages in macula retinas from AMD patients and control group. In the Retina Macula AMD samples, Siglecs-7, -9, and -11 all demonstrate stronger positive correlations with M0 and M1 signatures compared to M2 macrophages. Similarly, this result is observed for Siglec-7 and -9 in Retina Macula Control samples (**Figure 1B**, **Supplementary Figures 2A, B**). However, the correlation coefficient appears to dramatically drop for associations between Siglec-11 and M0/M1 signatures in the control samples compared to AMD samples (**Figure 1B**, **Supplementary Figure 2C**).

### PolySia-NPs bind to human Siglec-7, -9, -11 and mouse Siglec-E and macrophages

**Figure 2:**
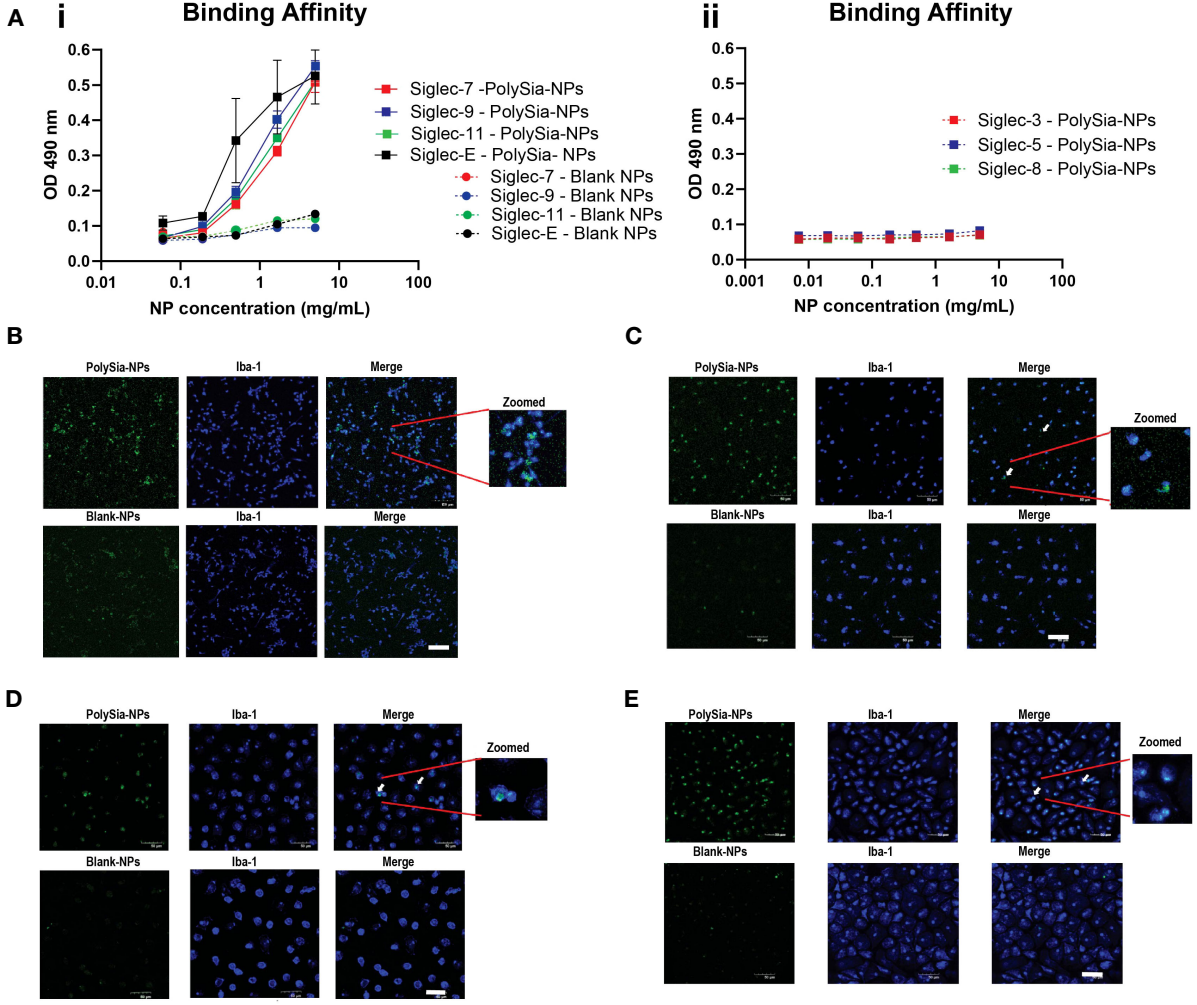
Binding assay of PolySia-NPs to Siglecs and macrophages. The NPs containing PEG binding to the Siglecs were detected using anti-PEG biotin/horseradish peroxidase (HRP) system according to manufacturer’s ELISA protocol **(A)**. PolySia-NPs (square shape) and a blank-NPs control (round shape) at different concentrations (0.2−10mg/mL) were assessed for binging to Siglec-7 (red), -9 (blue), -11 (green) and Siglec-E (black) (A-i) and Siglec-3, -5 and -8 (A-ii). Mean ± SEM. LPS activated THP-1 cells **(B)**, M0 macrophages **(C)**, M1 macrophages **(D)** or M2 macrophages **(E)** were incubated with FITC-conjugated PolySia-NPs or FITC-conjugated Blank-NPs controls(green). Macrophages were fixed and stained for marker Iba-1 (blue). The representative image shows macrophages imaged for FITC conjugated PolySia-NPs or blank-NPs in green (1st column), IBA1+ macrophages in blue (2nd column) and merged colors with zoomed image (3rd column). NPs: nanoparticles. 50μm scale bar. Magnification: 100x.

PolySia-NPs were prepared using a core of polyethylene glycol (PEG) and poly-lactic co-glycolic acid (PLGA) copolymers to which polysialic acid (PolySia) was covalently attached (). To quantify nanoparticles binding to Siglecs, we used an ELISA-type binding assay where the specific binding capability of the PolySia-NPs to the extracellular domain of Siglecs -7, -9 and -11 immobilized via a C-terminal Fc tag was evaluated. The results showed that binding of PolySia-NPs significantly increased with the concentration of Siglecs -7, -9, -11 and mouse Siglec-E [orthologue mouse Siglec for human Siglec-9 (42)] compared to Blank-NPs (control lacking PolySia; **Figure 2A**i), showing that binding is specific to these Siglecs as expected. PolySia-NPs did not showed binding to Siglec-3, -5 or -8 (**Figure 2A**ii). In addition, a competitive binding assay revealed that binding of Siglec-7 Fc and Siglec-9 Fc proteins to sialic acid expressing Panc-1 cells was inhibited by PolySia NPs (50% reduction of Siglec-9 binding at 0.5 mg/mL and 28% reduction of Siglec-7 binding at 0.25 mg/mL (*) (**Supplementary Figure 2D**). This result indicates that PolySia-NPs bind to Siglec-7/9 Fc, thus inhibiting binding of the latter to Panc-1 cells.

Furthermore, to aid tracking of the NPs, a FITC conjugated PLGA was incorporated into the NP and their ability to bind to activated macrophages derived from a stimulated THP-1 human monocyte cell line or PBMC primary cells was examined. The primary PBMC-derived macrophages were cultured in polarizing conditions (M0, M1, M2). Macrophages were then treated with either FITC-conjugated PolySia-NPs or blank-NPs for 24h. FITC-Blank-NPs are nanoparticles without the PolySia, ligand selected as a suitable control. We observed co-localization of the FITC-conjugated PolySia-NPs with the macrophage/microglial marker Iba-1 in macrophages derived from LPS-activated THP-1 cells, and with activated PBMC derived macrophages (**Figure 2B–E**). The merged confocal images (**Figures 2B–E**, top row) show that FITC-conjugated PolySia-NPs bind to all macrophage populations and control “blank” NPs do not bind (**Figures 2B–E**, bottom row). FITC-Blank-NPs are nanoparticles without PolySia that can be taken up by phagocytic cells like macrophages and could be observed as FITC positive cells. However, the number and intensity of positive cells with FITC-Blank-NPs is clearly lower than that observed with FITC-PolySia-NPs.

### PolySia-NPs are not cytotoxic to macrophages

We analyzed the viability of THP-1 cells and PBMCs-derived M1-macrophages treated with the PolySia-NPs by using an MTT assay. THP-1 cells or PBMC-derived M1 macrophages were incubated in the presence of the PolySia-NPs or the Blank-NPs at 50 to 750 μg/mL range in a 96-well plate (**Supplementary Figure 3A**). Our results did not show a significant decrease in viability at any of the PolySia-NPs doses tested on THP-1 cells and PBMCs-derived M1-macrophages (**Supplementary Figures 3A-C**).

### PolySia-NPs suppress release of IL-1β, TNF-α, VEGF and IL-6, while increase IL-10 release from activated human macrophages

In order to validate the *in vitro* studies in human macrophages, we determined the expression of Siglec-7, -9 and -11 in M0, M1 and M2 monocyte derived macrophage (MDM) and evaluate the effect of oxidized (Ox) LDL and LPS *in vitro* on M1 macrophages. Flow cytometry analysis of Siglec-7, -9 and -11 expression on M0, M1 and M2 monocyte derived macrophage (MDM) from 3 healthy donors (N=3) showed high expression of Siglec-7 and -11 in M1-MDM compared to M0-MDM and monocytes (red dash line), while Siglec-9 is higher compared to M0 macrophages. High expression of Siglec-7 and -9 on M2-MDM was observed (**Supplementary Figures 4B, C**).

**Figure 3:**
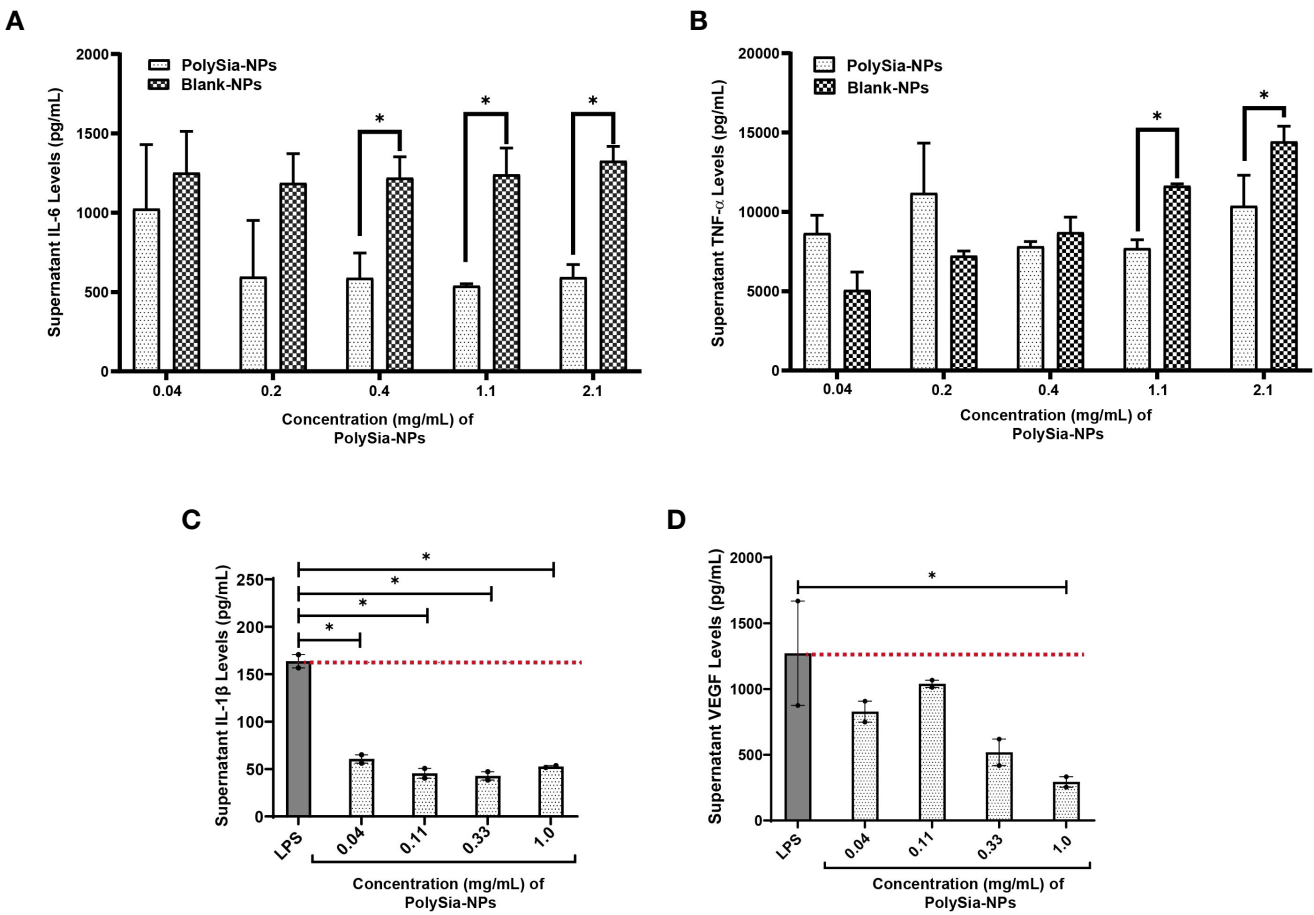
Cytokine measurement from human PBMCs-derived M1 macrophages supernatant. TNF-α **(A)** and IL-6 **(B)** protein concentration were determined by ELISA assay from M1 macrophages supernatant treated with serial dose range of PolySia-NPs and blank-NPs (0.04−2.1 mg/mL) in presence of LPS overnight. IL-1β **(C)**, VEGF **(D)** was also measured in M1 macrophages treated with a serial dose of PolySia-NPs (0.01−1 mg/mL) overnight. LPS served as a positive control. Mean ± SEM. *p<0.05.

The Functional effects of PolySia-NPs on macrophages were investigated by evaluating alterations in secreted pro- and anti-inflammatory cytokines. TNF-α and IL-6 were determined in M1 macrophages supernatant after culturing purified monocytes from healthy donors in M1 polarizing conditions and treating with PolySia-NPs or Blank-NPs in a range of 0.04-2.1mg/mL. The results showed a significant decrease of both TNF-α and IL-6 at high concentration (1.1 and 2.1mg/mL) of PolySia-NPs when compared to blank-NPs and IL-6 at 0.4 to 2.2mg/mL compared to blank-NPs (**Figures 3A, B**). In addition, IL-1βand VEGF release was determined by ELISA from M1 macrophages supernatant following culture of PBMCs from healthy donors under M1 polarizing conditions and treatment with PolySia-NPs at range of 0.04 -1 mg/mL. We cover this range of NPs concentrations to ensure effectiveness when is used on *in vivo* models. The results showed that PolySia-NPs at 0.11, 0.33, and 1 mg/mL led to a decrease of IL-1β by 3.58-, 3.81-, and 3.10-fold respectively, compared to LPS (p < 0.05). Similarly, released VEGF levels following incubation with PolySia-NPs at 1 mg/mL fell 4.33-fold compared to the LPS group (p < 0.05) (**Figures 3C, D**).

THP-1 cells were differentiated to macrophages with PMA (10 ng/mL) and treated with PolySia-NPs at 0.04−1 mg/mL. A significant reduction of TNF-α release occurred at 0.33 mg/mL, 3.41-time fold decrease of total solids (p < 0.01) and an increased IL-10 by 1.46-time fold with PolySia-NPs at 0.33 mg/mL, when compared to LPS incubation only (p < 0.01). Similarly, 1 mg/mL of PolySia-NPs showed significant 2.11-time fold reduction of TNF-α (p < 0.05) and increased IL-10 by 1.49-time fold compared to treated with LPS only as control (p < 0.01) (**Supplementary Figures 5A, B**).

In addition, we evaluated the effect of oxidized (Ox) LDL to activate macrophages in presence of PolySia-NPs. The results showed no cytotoxicity effect of PolySia-NPs in a range of 1.0 to 0.011mg/mL concentration in OxLDL treated macrophages (**Supplementary Figure 5C**). PolySia-NPs showed dose-depending inhibition in TNF-α production in OxLDL and LPS treated macrophages when compared with treated with 10% sucrose vehicle (red dash line) with significant decreased at 1. 0 and 0.33mg/mL concentration (p<0.05 and p<0.001, one-way ANOVA) (**Supplementary Figure 5D**).

### PolySia-NPs are safe and well tolerated in the eye

The safety and tolerability profile of PolySia-NPs was evaluated *in vivo* using mice and rabbits. PolySia-NPs were injected intravitreally (IVT) and compared to PolySia alone or Blank NPs controls to evaluate for clinical signs of damage or inflammation.

Overall, PolySia-NPs were well-tolerated in mice after IVT injection. There were no visible morphological changes were found in any group upon ocular examination at 14 days after IVT injection (**Supplementary Figures 8A, B**). Intraocular pressure (IOP) was slightly decreased at 4h and on days 1 and 2 post IVT injection. IOP recovered to the baseline on day 3 (**Supplementary Figure 8C**). The b-wave amplitude remained unchanged over the time and a-wave was lower in all treated groups compared to the baseline (**Supplementary Figure 8D**). H&E staining on retinal tissue sections did not show significant histological changes between the groups (**Supplementary Figure 8E**), in line with OCT data for total retinal, ONL or INL thickness in all the groups at day 14 compared to baseline (**Supplementary Figure 8F**).

In addition, IVT injection of PolySia-NPs in rabbits was also evaluated for safety. Overall, The IVT inoculation of PolySia- NPs at low, medium and high concentration/eye was well tolerated without apparent negative effects. Animals gained a normal amount of body weight from average weight 1.7 ± 0.1kg at the baseline to 1.9 ± 0.1kg over the course of the study (**Supplementary Figure 9A**). All baseline OE scores were 0. On Day 1, some eyes had mild inflammation (OE scores of 1-4), but on Day 3 and 7, all scores were 0. On Day 13, some eyes had mild inflammation (OE scores of 1) in vehicle Group and treated with the high dose of the NPs. All eyes remained within the normal IOP range for this strain and species at all timepoints measured. ERGs were used to evaluate retinal function, and the average amplitudes of both a-waves and b-waves were not found to differ between groups or timepoints. Optical Coherence Tomography imaging of the posterior section of the eye showed that the baseline mean ONL thickness of all groups was 32-35 μm (**Supplementary Figure 9B**), and it slightly decreased to 27-29 μm at the time of necropsy. Similarly, the intraocular pressure (IOP) didn’t change (**Supplementary Figure 9C**) and total retinal thickness of all groups was 179-187 μm at baseline and remained similar between 180 μm and 187 μm on Day 14 (**Supplementary Figure 9D**). Thus, the injection of PolySia-NPs into the intravitreal space can be considered nonadverse, as there were only minimal to mild signs of inflammation and no associated/secondary changes observed in other ocular structures.

### PolySia-NPs reduced outer nuclear layer thinning and decreased macrophage infiltration in the bright light damage mouse model

**Figure 4:**
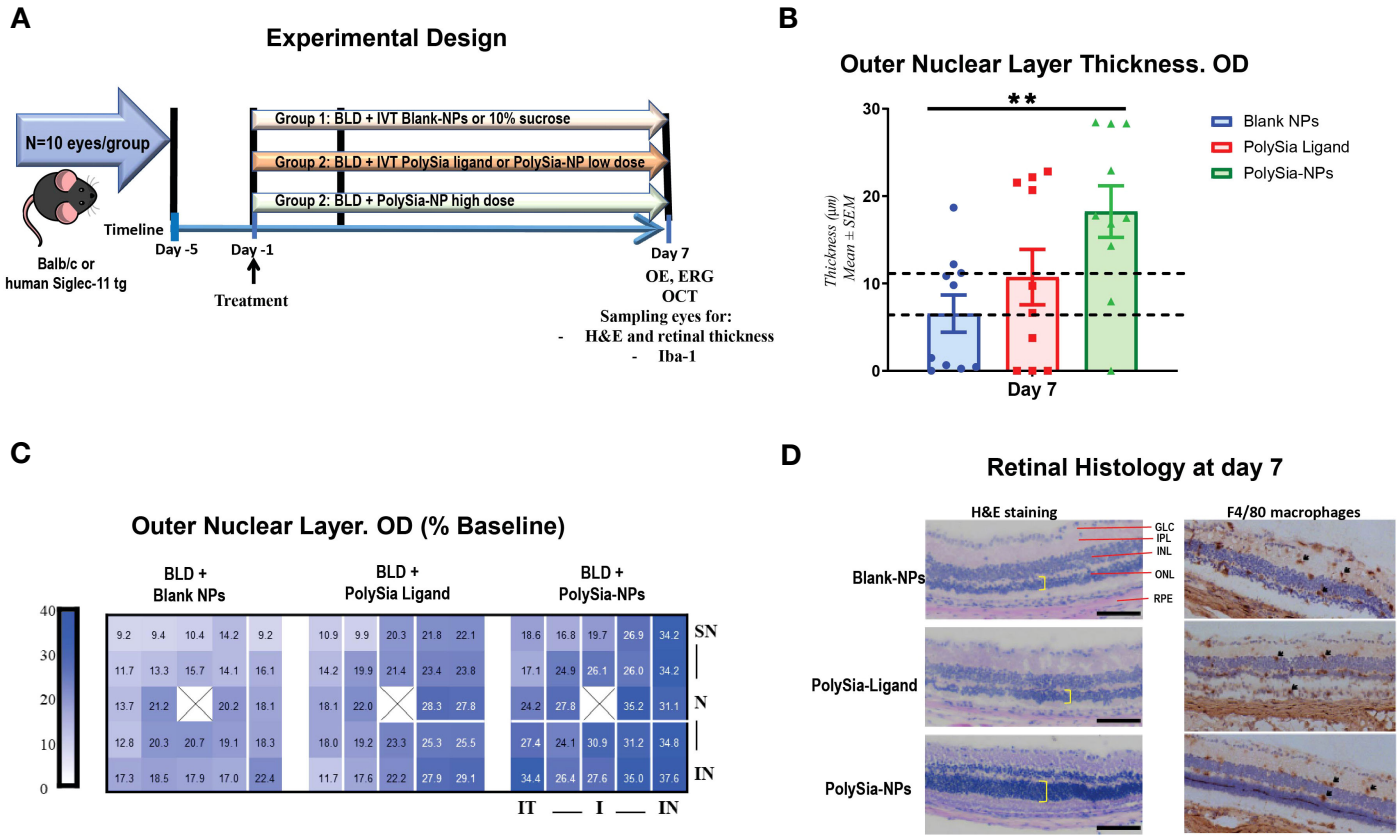
*In vivo* bright light damage (BLD) murine model clinical assessments. Experimental design for the bright light damage (BLD) model on Balb/c mice or humanized Siglec-11 tg mice **(A)**. Outer Nuclear Layer (ONL) thickness determined by SD-OCT at Day 7 after BLD. Mice were treated with PolySia-NPs, PolySia ligand alone or Blank NPs. Data are presented as % of the baseline values from 11 mice per group and 10 for Blank-NPs. The dotted lines represent the usual damage seen in naïve animals induced using this same protocol (6.6-12 µm). **(B)**. Heat map of ONL thickness across the 25 grid points. The value of each spot is the represented value. X denotes the optic nerve; SN, superior nasal; IN, inferior nasal; IT, inferior temporal, N, nasal **(C)**. Retinal H&E and macrophage (F4/80) immunostaining **(D)**. Black arrows indicate macrophages. GLC, Ganglion Cell Layer; IPL, Inner Plexiform Layer; INL, Inner Nuclear Layer; ONL, Outer Nuclear Layer (yellow shows thickness); RPE, Retinal Pigmented Epithelium/Bruch’s Membrane Choroid; NPs, nanoparticles. Mean ± SEM. **p<0.01. OE, Ocular examinations; ERG, Electroretinography; OCT, Optical coherence tomography.

Data showed that mice treated with PolySia-NPs prior to exposure to high intensity light had less loss of the ONL of the retina assessed by Spectral Domain Optical Coherence Tomography (SD-OCT), compared to animals treated with Blank NPs (p=0.0028) (**Figure 4A**). The group treated with free PolySia ligand alone did not show significant difference compared to Blank-NP. The dotted line represents the usual damage and above that line is considered a significant clinical improvement (**Figure 4B**). Heat map correlations indicate relative changes of the retinal layers as measured by OCT centered on 3 different regions of the eye (**Figure 4C**). The darker intensity of the blue square at the superior orientation (SN) in the PolySia-NPs treated mice showed significant maintenance of the ONL in that region compared to the ONL in the same region in mice treated with PolySia only or Blank-NPs (**Figure 4C**). Under physiological conditions, the ONL comprises approximately 35% of the total retina, therefore, any protective effect on ONL thickness conferred by pharmacological intervention is typically not seen as a statistically significant difference in total retinal thickness. In line with OCT data, H&E staining revealed a less of a ONL thickness in the PolySia-NPs treated mice compared to mice treated with PolySia only or Blank-NP. Also, immunohistochemical (IHC) analysis of the retina revealed a decreased macrophages (F4/80 immunoreactivity) only in PolySia-NPs treated eyes after BLD-induction, suggesting that unconjugated ligands are not effective in protecting the retina (**Figure 4D**). While the F4/80 macrophage counting in the retina did not reach statistical significance (**Supplementary Figure 6B**), the images clearly illustrate that the extent of retinal damage is greater in the blank-NPs and PolySia ligand-treated groups compared to the PolySia-NPs group. The latter group exhibited fewer damages and better-preserved retina layers (**Supplementary Figure 6A**).

**Figure 5:**
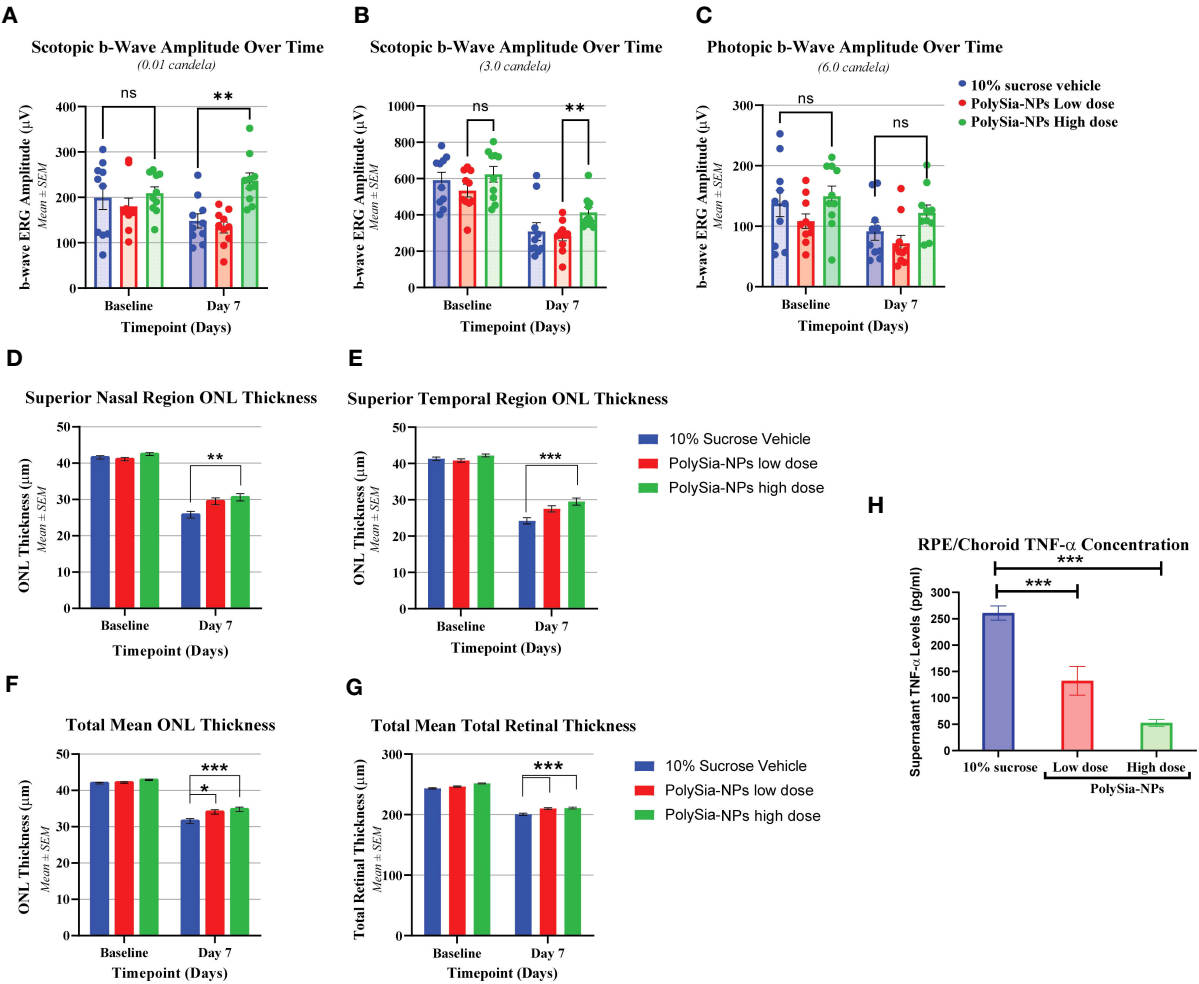
*In vivo* bright light damage (BLD) model in humanized Siglec-11 transgenic mice clinical assessments. Electroretinography (ERGs) were performed at Baseline and on Day 7 after bright light damage (BLD). b-wave amplitude over the time were recorded in mouse treated with 10% sucrose (vehicle), PolySia-NPs low (red) and high dose (green) **(A-C)**. Outer Nuclear Layer (ONL) thickness at Baseline and at Day 7 after bright light damage (BLD) of superior nasal **(D)** and temporal region **(E)**, and total mean ONL and retina thickness **(F, G)** N=89-90. Cytokine quantification in ocular tissues from bright light damage (BLD) model. TNF-α concentration (pg/mL) on RCS (RPE/Choroid/Sclera) treated with vehicle (sucrose 10%), and PolySia-NPs at low and high dose/eye **(H)**. Mean ± SEM. *p<0.01, **p<0.001, ***p<0.0001 by 2way ANOVA statistical analysis. NPs, nanoparticles; NS, no significant.

Another *in vivo* bright light damage (BLD) model was investigated in humanized Siglec-11 transgenic mice. Ocular examination (OE) and electroretinography (ERGs) were assessed with PolySia-NPs at baseline and at day 7 after bright light damage (BLD). This transgenic mouse expressed human Siglec-11 and mouse Siglec-E (**Supplementary Figure 7**) making it possible to study the effect of this Siglecs *in vivo*. At baseline and on Day 6, all OEs were normal, with no other observations. During the scotopic 0.01 cd measurement, the b-wave amplitude in all groups was similar at baseline. On Day 7, the b-wave amplitudes of PolySia-NPs in the high dose treated group was higher than the other groups (p<0.01) and similar to the baseline (**Figure 5A**). During the scotopic 3.0 cd measurement on Day 7, the b-wave amplitude of the groups was decreased compared to baseline, but similar between groups; while, with the b-wave amplitude of PolySia-NPs treated at high dose was significantly higher compared to PolySia-NPs at low dose (p<0.05) (**Figure 5B**). During the photopic 6.0 cd measurement, the b-wave amplitude of all groups was similar at baseline. On Day 7, the b-wave amplitude in all groups was lower compared to baseline with the b-wave amplitudes in PolySia-NPs at high dose was slightly higher but not significant (**Figure 5C**).Outer nuclear layer (ONL) thickness was assessed using regional OCT analysis at baseline and day 7. On Day 7, there was a significant decrease in ONL thickness from baseline in all groups and regions. However, the decrease in ONL thickness was more pronounced in the superior regions (nasal and temporal) for the eyes treated with the vehicle compared to those treated with PolySia-NPs on Day 7 (p<0.0001, p<0.001; **Figures 5D, E**) (N=89-90 readings). In addition, the mean total ONL thickness and mean total retinal thickness were increased with both low and high doses of PolySia-NPs compared to sucrose-vehicle treated eyes (p<0.0001, p<0.01; **Figures 5D–G**) (N=89-90 readings).

Finally, cytokine quantification in ocular tissues showed that TNF-α on RCE (RPE/Choroid/Sclera) decreased significantly in a dose-dependent manner by 2-fold and 5-fold change after treatment treated with PolySia-NPs at low and high dose/eye respectively, compared to vehicle (sucrose 10%) (**Figure 5H**).

### PolySia-NPs reduce laser-induced choroidal neovascularization lesion size

**Figure 6:**
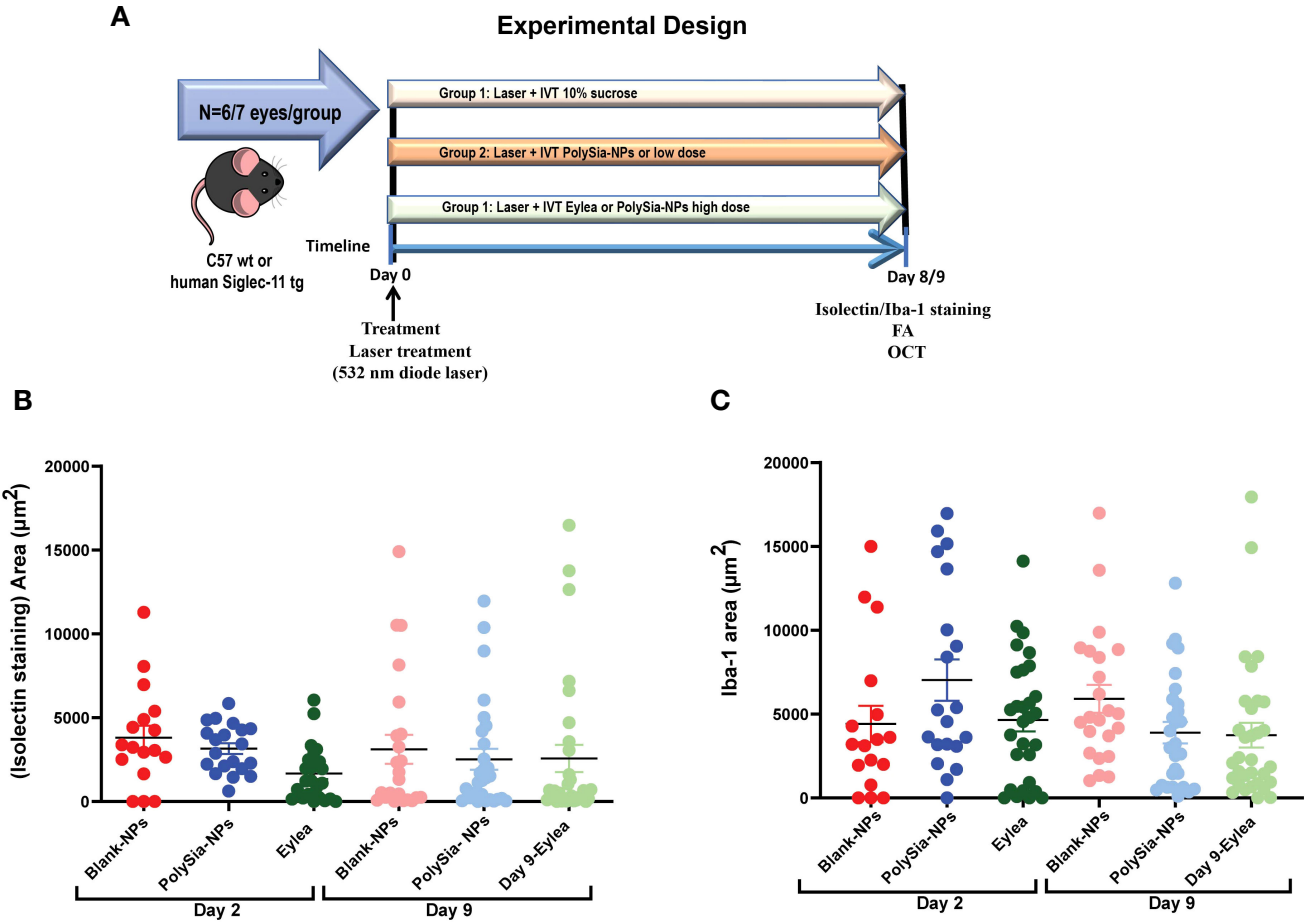
Experimental design for the Laser-induced choroidal neovascularization (CNV) model on C57 wt mice or humanized Siglec-11 tg mice **(A)**. The CNV lesion size and macrophage/microglia activation determined by isolectin-B4 and Iba-1 staining in C57BL/6 mice retinas. Three laser lesions were placed in the right eye around the optic nerve head using a 532 nm diode laser. CNV lesions or macrophage/microglia were determined by isolectin-B4 **(B)**, or Iba1 **(C)** staining respectively. Quantification of the area (μm^2^) in wholemount retinas after IVT treatment with PolySia-NPs or blank-NPs and compared to Eylea (anti-VEGF) at 80 mg/mL, 2 and 9 days after CNV induction. Mean ± SEM. FA, Fluorescein Angiography; OCT, Optical coherence tomography.

The laser-induced CNV model is a VEGF-driven model, in which VEGF initially is secreted by injured RPE cells via the HIF-1α pathway and reactive oxygen species (51, 52), and it also behaves as a chemoattractant (53). In the first 2 days after lesion induction, neovascularization is initiated by VEGF produced by these alternate mechanisms. It then transitions to VEGF produced by the VEGF-recruited monocytes and activated microglial cells (54). Therefore, direct inhibition of VEGF during the first 2 days is more effective than suppression of monocyte/microglial activation, explaining why the anti-VEGF therapy (Eylea), a VEGF inhibitor, is effective even on Day 2 (**Figure 6**). To evaluate the effectiveness of PolySia-NPs in inhibiting CNV lesion size driven by VEGF we evaluated early at day 2 and later at day 9 (**Figure 6A**). It is hypothesized that the inhibition of neovascularization by PolySia-NPs in this VEGF-driven model is through the suppression of activated VEGF-secreting monocytes/microglial. The results (mean ± SEM) showed that PolySia-NPs treated eyes had decreased the CNV lesion area at day 9 determined by isolectin-B4 staining compared to Blank NPs treated (2520 ± 620 vs 3112 ± 868 μm^2^, p=0.57) and like anti-VEGF Eylea treated (2,520 ± 620 vs 2,570 ± 809 μm^2^, p=0.96) at Day 9 (**Figure 6B**). Similarly, the Iba-1 staining for macrophages/microglia revealed similar sized areas compared to the anti-VEGF Eylea treated group (3894 ± 636 vs. 3744 ± 734 μm2, p=0.88) (**Figure 6C**).

**Figure 7:**
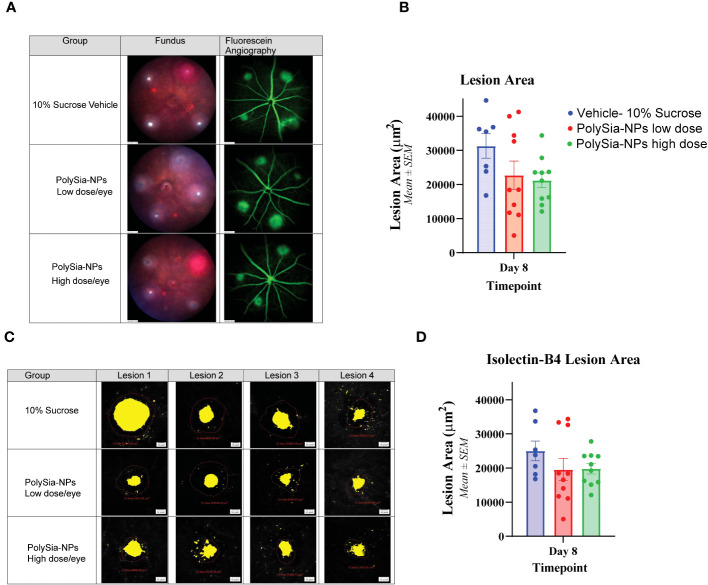
Laser-induced choroidal neovascularization (CNV) model in humanized Siglec-11 transgenic mice. The mice’s eyes received a single IVT dose of either PolySia-NPs at low and high dose/eye or a 10% sucrose vehicle. After the treatment, CNV was induced by creating four single laser spots OU surrounding the optic nerve using a 532 nm diode laser. The representative micrographs show the lesion size from fundus imaging and fluorescein angiography **(A)** and retinal flat mount immunohistochemistry staining for Isolectin-B4 **(B)** on Day 8. The lesion size was evaluated and quantified at Day 8 for fluorescein angiography **(C)** and by Immunohistochemistry Staining with Isolectin-B4 **(D)**.

In the CNV study in Siglec-11 humanized mice, the size of the lesion was assessed by using fluorescein angiography and Isolectin-B4 staining. The results showed that on Day 8, the control group had the largest lesion area (5,269.8 μm2), whereas treatment with PolySia- NPs at low and high dose reduced the lesion size to 4,352.7 and 3,496.0 μm2; respectively (**Figures 7A, B**). Flat mount immunohistochemistry (IHC) staining of isolectin-B4 showed that the control group had the largest lesion area (25,026.8 μm^2^) that is reduced in the PolySia-NPs treated group at low and high doses to 19,573.6 μm^2^ and 19,818.9 μm^2^ respectively (**Figures 7C, D**).

## Discussion

In this study, we have developed a novel sialic acid mimetic nanoparticle platform for inhibiting macrophage activation during development of macular degeneration. Unlike strategies that primarily aim to inhibit neovascularization or complement (20), we employed a nanoparticle platform with PolySia as ligand to target Siglecs, thereby inhibiting macrophage-mediated responses and suppressing inflammation and vascularization. We determined by analyzing published RNA-seq data, that Siglecs expression increased in the macula region of retinas associated with AMD. Transcriptomic analyses confirmed the overexpression of Siglecs -7, -9, and -11 in the macular region of AMD retinas. Additionally, there is a significant change with increased expression of Siglec-14 and -15 in retinas associated to AMD. Siglec-14 and -15 are expressed on myeloid cells associated with the activating adapter protein DAP12 that promotes activation. However, there is limited knowledge regarding the role of Siglec-14 and -15 in macrophages in the context of inflammation associated to AMD. Further exploration of these Siglecs as potential targets for AMD treatment would be necessary.

Analysis of the RNAseq data from the clinical AMD dataset previously published showed expression of Siglecs-7, -9, and -11 in M0, M1 and M2 macrophages with moderate to strong correlations between Siglecs-7, -9 and M0 and M1 macrophages compared to low correlation in M2 macrophages, suggesting that Siglecs play a key role in keeping resting M0 macrophages from activating and to tightly regulate acutely activated M0, and M1 proinflammatory macrophages in AMD. In addition, Siglec-11 shows a significantly low correlation with M0 and M1 macrophages in normal patients, suggesting that Siglec-11 expression in macrophages is associated to AMD patients. These results suggest the increased presence of expressed Siglecs in activated macrophages, particularly M1, plays a crucial role in AMD responses. This enhanced expression of Siglecs, a marker of activated inflammatory cells, in AMD patients supports the critical role of inflammation in AMD and potential role of macrophages as determinants of AMD phenotype. To investigate binding to activated macrophages, FITC-tagged PolySia-NPs were used, and binding to M1 and M2 macrophages was demonstrated with a pattern primarily located on the outer membrane of fixed cells. This suggests that PolySia-NPs are binding to sialic acid binding lectin such as Siglecs (28, 30, 55). These observations led us to test the biological effects of PolySia-NPs on activated macrophages.

The *in-situ* binding assessment demonstrated the binding of PolySia-NPs to specific human Siglecs including Siglec -7, -9, -11 and to mouse Siglec-E, which is the orthologous counterpart of human Siglec-9 but not to Siglec-3, -5 and -8. Blank NPs, which were not decorated with PolySia, and did not show any binding to the Siglec receptors was used as control. This demonstrated that the PolySia- NP binding to Siglecs is glycan-mediated through PolySia. Moreover, competitive binding assays confirmed the specific binding of PolySia-NPs to Siglecs.

PolySia-NPs were tested *in vitro* on human monocytes and activated macrophages to determine the ability of PolySia-NPs to resolve the pro-inflammatory macrophage activation. When M1 macrophages were treated with PolySia-NPs, proinflammatory TNF-α, IL-1β and IL-6 cytokines decreased, while anti-inflammatory and pro-resolution cytokine IL-10 increased. This suggests a repolarization of the macrophages from the pro-inflammatory M1 to the resolution M2c state. This agrees with previous findings on human THP-1 macrophages where it has been demonstrated that PolySia-NPs attenuate proinflammatory activation of human monocytes (43, 56). The decreased in VEGF production on macrophages observed in the presence of PolySia-NPs suggests the polarization of M2d state to the resolution M2c state. Additionally, we compared the effect of activation with OxLDL as it is a more representative macrophage agonist seen in AMD. The results showed similar inhibitory effect of TNF-α production by PolySia-NPs in presence of OxLDL compared to LPS treated macrophages, validating our data obtained with LPS. Thus, PolySia-NPs emerged as a strong candidate to ameliorate the inflammation and vascularization caused by recruited macrophages in AMD by decreasing TNF-α, IL-6, IL-1β and VEGF, while upregulation of IL-10 promotes tissue healing and regeneration (15, 57).

Nanoparticles have been evaluated in various cell and animal models and demonstrated no or very mild inflammatory reactions but have never been shown to reduce cytokines production on their own (41, 58, 59). We carefully assessed the *in vivo* safety of IVT injections of PolySia-NPs in mice and rabbits with no sign of any clinical or histological toxicity, validating the platform safety for retinal injection. To test how important inflammation, in particular macrophage inflammation, in this AMD model we injected intravitreal PolySia-NPs in the BLD mouse model. After inducing BLD, these mice can be expected to lose 80% of their photoreceptor layer with ocular expression of proinflammatory and chemotactic cytokines including IL-1β, chemokine (C-C motif) ligand 2, cyclooxygenase-2, and TNFα-genes (60). The first few days of the BLD model photooxidation occurs resulting in the induction of apoptosis. The recruitment of peripheral blood phagocytic macrophages results in the outer nuclear layer cell loss. Furthermore, recent studies on BLD models suggests that infiltrating M1 macrophages mediate retinal cell loss similar to what is seen in AMD pathology making the BLD model clinically relevant (10). This finding reinforces the suitability of using the BLD model to study the effectiveness of PolySia-NPs in clinically treating AMD.

We demonstrated that treatment with PolySia-NPs reduced the loss of retina ONL thickness in eyes after being exposed to high intensity light and showed decreased macrophage infiltration compared to eyes treated with PolySia alone or Blank-NPs. Similarly, the decreased macrophage infiltration in PolySia-NPs treated eyes suggests that the NP scaffold is required to enhance the activity of PolySia to target Siglecs and effectively elicit an anti-inflammatory/protective response. It was previously observed that disialic acid NPs ameliorated inflammatory responses compared to free disialic acid ligand in line with our results and thereby validating the nanoparticle-based platform as necessary therapeutic approach (42). The PolySia-NPs not only showed decreased macrophages recruitment, but also a decreased TNF-α and IL-6 and IL-1β in ocular tissues from the BLD model consistent with our *in vitro* findings in macrophages. These findings reinforce the importance of macrophage activation in this clinically relevant model of advanced dry macular degeneration, it also confirms the ability of PolySia-NPs to down modulate pro-inflammatory macrophages significantly *in vivo*.

The main pathway for how high energy visible light activates macrophages is likely from intense the direct effect of the light and production of oxidative byproducts like what is seen in the pathogenesis of AMD. The direct retinal exposure to high energy visible and ultraviolet light on the retina upregulates heat shock proteins (HSP). HSP 60 binds to the surface of CD14 and toll like receptor including TLR-4 in similar fashion to how LPS binds to TLR-4 (56). Retinal cells express multiple TLR family members, and the retinal pigment epithelium (RPE) is particularly rich in TLR expression. Reports linked TLR4 polymorphisms to AMD susceptibility, and the role of TLR agonists (e.g. DAMPs) and the outcomes in age-related macular degeneration (AMD) have been well studied in particular the role of Carboxyethyl pyrrole (CEP) (61, 62). CEP has been shown to be a ligand for TLR-2 and has been implicated in promoting angiogenesis in addition to being pro inflammatory (63). The production of oxidized cholesterol esters and oxidized phospholipids are ligands for TLR 4 that activate macrophages in similar manner as LPS activation, and are involved in the patho-mechanism of AMD and the BLD model (64). Scavenger receptor CD36 binds oxidized, acetylated LDL and oxidized phospholipids but not native LDL and these oxidative byproducts play a major factor in the development of both AMD and the BLD model (64–66).

In addition, we tested the PolySia-NPs in the BLD model in Siglec-11 humanized mice to determine the functional changes in preventing photoreceptor death by inhibiting only macrophages and to rule out the potential role of the adaptive immune system. ERG readings and ONL thickness. Human Siglec-11 is expressed selectively in microglia and tissue macrophages, specifically binds to α2–8-linked sialic acids like polysialic acid (33, 67). Given the lack of a mouse ortholog in genome databases (59), we utilized humanized Siglec-11 transgenic mice to circumvent this issue by knocking in human Siglec-11. The BLD study revealed that exposure to blue light caused a significant decrease in the b-wave amplitude of mice, indicating severe photoreceptor damage. After administering a high dose of PolySia-NPs, significant improvements in the b-wave amplitude were observed, and OCT scans indicated a recovery in retinal thickness. These findings suggest that PolySia-NPs treatment can not only prevent cell loss but neuroprotect photoreceptors to preserve visual function. Current complement depletion treatments have demonstrated only reduction in rate of retinal cell loss but have not demonstrated any improvement in visual function (68). The recovery of ERG amplitude in human patients would translate into visual function or acuity improvement. Ongoing, first in human administration in patients with central geographic atrophy and visual loss will test this hypothesis. Moreover, retinal thickness and other clinical outcomes could be significantly improved in treated AMD patients after multiples dose treatments over time. Here, our experimental design allowed us to test the effects of the PolySia-NPs after 1 dose injection only; when in patient’s treatments, the regime will be multiple injections for months.

We tested the PolySia-NPs in the *in vivo* laser-induced choroidal neovascularization (CNV) to evaluate its ability to resolve VEGF producing M2d macrophages and assess the effect on retinal neovascular lesion size, macrophage/microglia infiltration and magnitude of vascular leakage. The CNV model is well characterized and has been used to develop all current FDA approved treatments for exudative AMD. Like human disease, the immunopathogenesis and mechanism involves macrophages in initiating the CNV response through the expression of pro-angiogenic cytokines, such as vascular endothelial growth factor (VEGF) (12, 13, 69). Here the PolySia-NPs clearly showed reduction in lesion size and macrophage/microglia areas in C57BL/6 mice retinas compared to Blank-NPs and comparable to Eylea, a potent FDA approved anti-VEGF therapy for exudative AMD. These results indicate that the PolySia-NPs may have comparable effects to clinically utilized anti-VEGF therapies. Similarly, another study on Siglec-11 humanized mice showed reduction of the lesion size compared to Blank NPs and 10% sucrose vehicle, demonstrating similar *in vivo* effects on targeting either human or mouse orthologous Siglecs. In the murine laser-induced CNV model, there is a large infiltration of macrophages in the CNV lesions and associated to arteriolar CNV in old mice suggesting a key role in the pathobiology (9). Macrophages have been directly implicated in the early recruitment of cellular components to the incipient CNV and are involved in the initial development of neovessels via release of angiogenic factors like VEGF, as part of the inflammatory response in the lesion site (70–73). By targeting and agonizing Siglecs-expressing macrophages/microglia recruited into the lesion site during AMD, it is possible to modulate pro-inflammatory macrophage to prevent neovascularization and promote resolution of retinal CNV. Similarly, microglia expressing human Siglec-11 may interact with PolySia-NPs and reduce proinflammatory mediators like TNF-α in line with previous findings showing that PolySia on neurons interact with Siglec-11 to prevent neurotoxicity (33). Although our results suggest macrophage repolarization after Siglec agonism with PolySia-NP in the *in vivo* CNV model, further investigations need to be conducted to supports the hypothesis that macrophage polarization is the major determinant in AMD.

Intravitreal anti-vascular endothelial growth factor (VEGF) drugs have revolutionized treatment for neovascular age-related macular degeneration (NV-AMD). However, treatment for geographic atrophy which focuses on complement factor depletion strategies has yet to provide a visually significant benefit to these patients. The ability to modulate macrophage functions may prove to be a promising strategy that can provide the type of revolution that anti-VEGF therapies have realized. Furthermore, the ability to modulate macrophage with PolySia nanoparticles has raised the interest in using these small glycoengineered molecules to modulate immunologic functions in a variety of diseases (74–76). Our glycan-functionalized NPs platform can be suitable to carry multiple molecules with different solubilities, be easily functionalized, have excellent biocompatibility, and show great stability, making it a powerful and versatile tool as potential drug therapy and to better understand the role of different inflammatory cells in the pathogenesis of many different immune mediated diseases. The benefits of this platform is the ability to present sialic acid ligands in a multivalent fashion which is necessary for agonizing Siglecs to achieve better avidity and effectiveness in modulating immune cells (42, 77, 78). Glycan modulators of microglial/macrophage polarization like our PolySia-NPs has the potential to not only lead to a highly effective clinical therapy to treat AMD, but provides a robust tool for determining the relative role of different immune cells in these diseases (22).

In conclusion, PolySia-NPs are effective in inducing an anti-inflammatory response in macrophages by targeting certain CD33r Siglecs. These findings indicate that the intravitreal delivery of PolySia-NPs to the retina may be a viable therapeutic approach for treating retinal degenerative diseases by regulating the innate immune response. Moreover, the *in vivo* safety and efficacy studies have demonstrated the tolerability of IVT administration of glycan-functionalized nanoparticles in ocular tissue providing adequate FDA compliant data to allow the first ever nanoparticle glycomimetic immune resolving modulator to enter human clinical trials to treat patients with geographic atrophy secondary to AMD. This paper paves the way for the development of a novel class of immune modulating therapeutics that can treat para, chronic, and acute inflammatory disease with unmet medical need such as diabetic complications, neurodegenerative disease, fibrotic diseases, and cancer.

## Data availability statement

The datasets presented in this study can be found in online repositories. The names of the repository/repositories and accession number(s) can be found in the article/**Supplementary Material**.

## Ethics statement

The studies involving humans were approved by Clinical records and a family questionnaire were obtained for all donors. The studies using human tissues were performed in accordance with FDA regulations and the Eye Bank Association of America (EBAA) medical standards regarding utilization of human tissue. Written, informed consent was obtained from all donors who provided human samples in accordance with the guidelines of the Declaration of Helsinki for research involving the use of human tissue. The protocols for these studies were approved by the Pharma Repository Governance Committee at Genentech that serves as the Genentech/Roche Institutional Ethical Committee to ensure that research on human samples stored in Genentech bio-repositories is performed in accordance with the subject’s informed consent and with global ethical guidelines. PMID: 31995762. The studies were conducted in accordance with the local legislation and institutional requirements. Written informed consent for participation was not required from the participants or the participants’ legal guardians/next of kin in accordance with the national legislation and institutional requirements. All animals studies were performed by Experimentica Ltd., Vilnius, Lithuania and by Powered Research, Durham, NC, USA in accordance with ARVO Statement for the Use of Animals in Ophthalmic and Vision Research and the EC Directive 2010/63/EU for animal experiments. The studies involving animal participants were reviewed and approved by either the Animal Welfare Ethical Board of Lithuania or Finland (Experimentica Ltd. animal license number G2-151, ESAVI-010815-2020), or the Institutional Animal Care and Use Committee (IACUC) (Powered Research). The studies were conducted in accordance with the local legislation and institutional requirements. Written informed consent was obtained from the owners for the participation of their animals in this study.

## Author contributions

AK-performing *in vitro* experiments. Coordinating *In vivo* studies. Designing and executing experimental protocols. VS- Supporting *In vitro* assays and manuscript writing. DP – Performing experiments on macrophages (ELISA and Protein). AL- Formulation of Nanoparticles. MKG – Supporting and guiding *in vitro* studies. PS - Data curation, methodology, formal analysis and investigation, reviewing and editing. SG- RNA seq data analysis. CS - Data interpretation, and reviewing the manuscript. MT and MG– Designing and supporting experimental design both *in vivo* and *in vitro*, reviewing data and the manuscript. All authors contributed to the article and approved the submitted version.

## Supplementary material

Supplementary Figure 1 Transcriptomic analysis for Siglec-1, -2, -3, -4, -5, -6, -8, -10, -12, -14, -15 and -16 in retina tissues associated to AMD. Expression of Siglecs was determined on the macular region of the retina from AMD patients (red) compared to control (blue). Data analysis was performed based on data from publicly available (GSE135092) RNA-seq datasets.

Supplementary Figure 2 Correlation analysis between M0, M1 and M2 macrophage signatures and Siglec-7 **(A)**, -9 **(B)** and -11 **(C)** RNAseq expression from macula retina samples from AMD (top) and control (bottom) groups. The Spearman’s correlation coefficient (R) value and p value was determined. Competitive cell binding assay **(D)**. Competitive binding assay between PE-Siglec-7 Fc and -9 Fc antibodies and Sialic acid expressing Panc-1 cells was determined by flow cytometry by pre-incubating with PolySia-NPs and Blank-NP. * Maximum inhibition of the anti-Siglec-7 and -9 antibodies and Sialic acid expressing Panc-1 cells.

Supplementary Figure 3 Viability assay on macrophages. THP-1 cells derived macrophages **(B)** and human PBMCs-derived M1 macrophages **(C)**. Study summary **(A)**. Cells were treated with a serial dose range of PolySia-NPs (0.01−5 mg/mL for THP-1 and 0.01-1mg/mL for M1 macrophages) (-▴-) and Blank-NPs (-▪-) and cytotoxicity determined by the colorimetric MTT assay. Results are shown as % viability where media alone served as internal negative control (-♦-) (100% viable), and media with dimethyl sulfoxide (DMSO) served as positive control (-▪-) **(B)**. Mean ± SEM. * p<0.05.

Supplementary Figure 4 Siglec expression on monocyte derived macrophages (MDM). Monocytes were isolated from human PBMCs were stained for CD14 and CD45 to determine the purity **(A)**. The expression of Siglec-7, -9 and -11 was determined by flow cytometry on M0-MDM, M1-MDM and M2-MDM and compared to monocytes (red dash line) from 3 different PBMCs donors. Representative histograms for Siglec-7, -9 and -11 intensity on M0-MDM, M1-MDM and M2-MDM Mare showing (*top*) and geometric means from 3 normal donors (*bottom*) **(B, C)**. Mean ± SEM.

Supplementary Figure 5 Cytokine measurement from human THP-1-derived macrophages supernatant. TNF-α **(A)** and IL-10 **(B)** protein concentrations were determined by ELISA assay from THP-1 macrophages supernatant treated with serial dose range of PolySia-NPs (0.04-1.0 mg/mL) in presence of LPS overnight. LPS served as a positive control. Viability assessments on LPS vs OxLDL stimulated M1-macrophages determined by the colorimetric MTT assay. M1-MDM macrophages were treated with either LPS or OxLDL and serial dose range of PolySia-NPs (1.0-0.11mg/mL). Results are shown as % viability where media alone served as internal negative control (-♦-) (100% viable), and media with dimethyl sulfoxide (DMSO) served as positive control (-♦-) **(C)**. TNF-α was determine by ELISA on cell supernatant form M1-MDM macrophages treated with either LPS or OxLDL and serial dose range of PolySia-NPs **(D)**. Mean ± SEM. * p<0.05, **p<0.01.

Supplementary Figure 6 Immunohistochemistry (IHC) for F4/80 staining in retinas from BLD model. Three representative figures of retinas stained for F/80 macrophages from Blank-NP, PolySia ligand and PolySia-NPs treated groups in BLD mice. Black arrows show F/480+ macrophages in a representative square area **(A)**. Quantification of F/80 staining from Blank-NP (blue), PolySia ligand (red) and PolySia-NPs (green) treated retinas from BLD mice **(B)**. NPs: nanoparticles.

Supplementary Figure 7 Human Siglec-11 transgenic mice production. Overview of the Targeting Strategy showing the CAG promoter-Kozak-human SIGLEC11 CDS-rBG pA” cassette cloned into intron 1 of ROSA26 in a C57BL/6N mice by CRISPR/Cas-mediated genome engineering **(A)**. RT-PCR analysis showing the expression of human Siglec-11 and mouse Siglec-E in the transgenic hSiglec-11 mice and wild type C57BL/6N mice **(B)**.

Supplementary Figure 8
*In vivo* clinical and histological evaluations after intravitreal (IVT) injections in mice. Slit lamp images of whole eye, injection site at day 0 and 14, and optic nerve **(A)**, fundus images at day 0 and 14 **(B)**, Intraocular presure measurments at baseline, day 0 (baseline), 4h and day 1, 2, 3, 7 a 14 after injection with Blank-NPs (blue), PolySia-NPs (red) and sialic acid ligand (green) **(C)**. fERG a-wave and b-wave and after exposing to flash of light at 2, 15, 65 and 250 lux of intensity after injection with blank NPs (blue and purple), PolySia-NPs (red and orange) and PolySia ligand (green, black) at day 0 (baseline) and 14 respectively **(D)**. Histological sections (H&E) showing the retinal layers and thickness quantification of inner nuclear layer, outer layers and total retinal, **(E)** of the retina. OCT thicknesses at day 0 (baseline) and 14 after injection with blank-NPs (blue), PolySia-NPs (red) and PolySia ligand (green), measured from SD-OCT scans **(F)**. Analysis made by Two-way ANOVA, p>0.05 for all. RGC: Retinal Ganglion Cell Layer, IPL: Inner Plexiform Layer, INL: Inner Nuclear Layer, ONL: Outer Nuclear Layer, RPE: Retinal Pigmented Epithelium/Bruch’s Membrane Choroid. White bar: 100μm. Data is presented as mean ± SEM. NPs, nanoparticles.

Supplementary Figure 9 Clinical evaluations after intravitreal (IVT) inoculations in rabbits at baseline and at day 14 after injection with vehicle (blue) or PolySia-NPs at low (red), medium (green) and high (magenta) dose. Change in Body Weight Over Time **(A)**. Optical Coherence Tomography (OCT) imaging of the posterior section of the eye. Change in Outer Nuclear Layer Thickness **(B)** Intraocular Pressure **(C)** and Total Retina thickness over the time **(D)**. NPs, nanoparticles.
