# Supplementary figures and images for: PolySialic acid-nanoparticles inhibit macrophage mediated inflammation through Siglec agonism: a potential treatment for age related macular degeneration
Source: Front Immunol. 2023 Nov 16;14:1237016. doi: 10.3389/fimmu.2023.1237016 (PMC10690618; doi:10.3389/fimmu.2023.1237016)

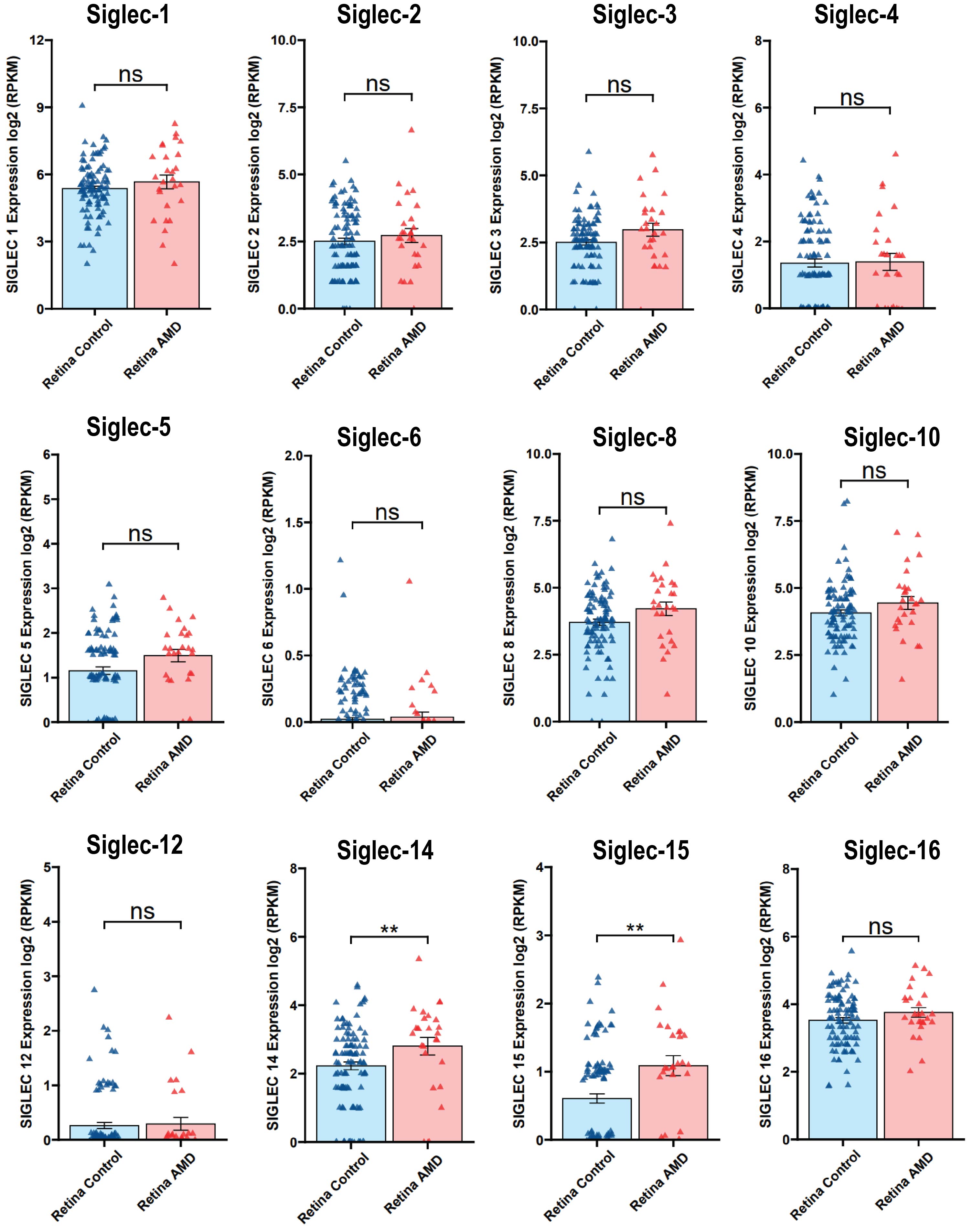

Supplement: Supplementary Figure 1 — Transcriptomic analysis for Siglec-1, -2, -3, -4, -5, -6, -8, -10, -12, -14, -15 and -16 in retina tissues associated to AMD. Expression of Siglecs was determined on the macular region of the retina from AMD patients (red) compared to control (blue). Data analysis was performed based on data from publicly available (GSE135092) RNA-seq datasets. [file Image_1.jpeg]

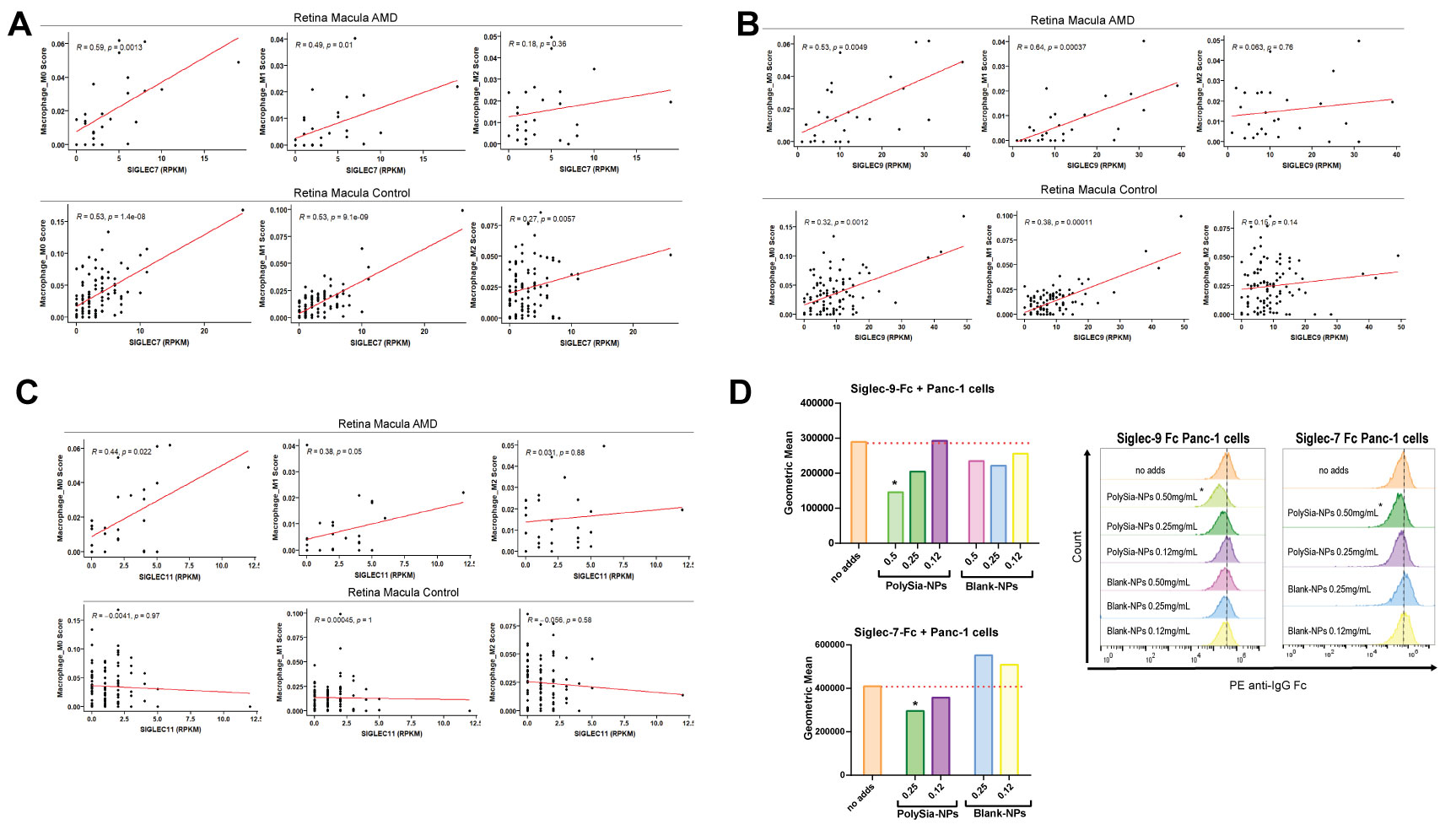

Supplement: Supplementary Figure 2 — Correlation analysis between M0, M1 and M2 macrophage signatures and Siglec-7 (A), -9 (B) and -11 (C) RNAseq expression from macula retina samples from AMD (top) and control (bottom) groups. The Spearman’s correlation coefficient (R) value and p value was determined. Competitive cell binding assay (D). Competitive binding assay between PE-Siglec-7 Fc and -9 Fc antibodies and Sialic acid expressing Panc-1 cells was determined by flow cytometry by pre-incubating with PolySia-NPs and Blank-NP. * Maximum inhibition of the anti-Siglec-7 and -9 antibodies and Sialic acid expressing Panc-1 cells. [file Image_2.jpeg]

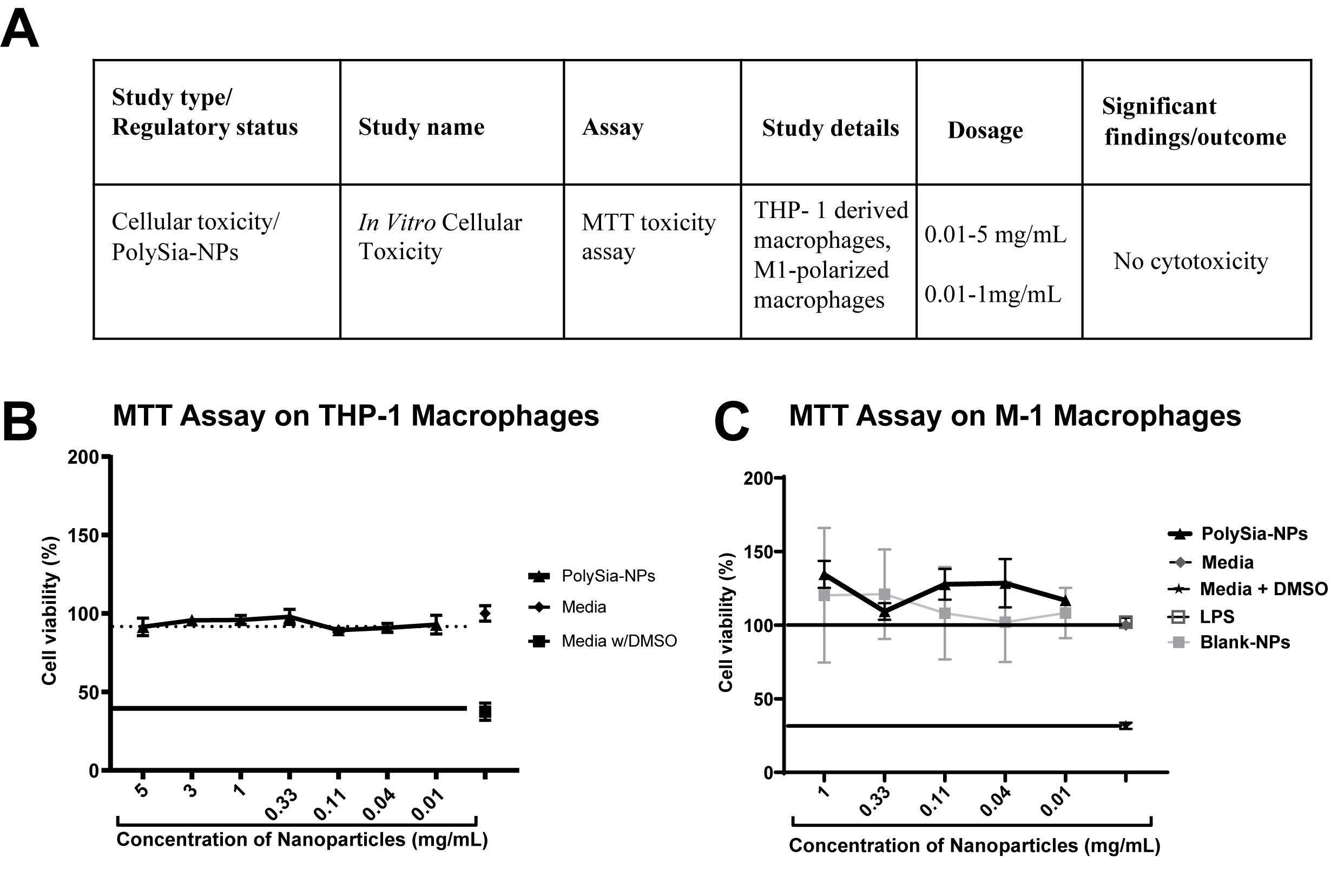

Supplement: Supplementary Figure 3 — Viability assay on macrophages. THP-1 cells derived macrophages (B) and human PBMCs-derived M1 macrophages (C). Study summary (A). Cells were treated with a serial dose range of PolySia-NPs (0.01−5 mg/mL for THP-1 and 0.01-1mg/mL for M1 macrophages) (-▴-) and Blank-NPs (-▪-) and cytotoxicity determined by the colorimetric MTT assay. Results are shown as % viability where media alone served as internal negative control (-♦-) (100% viable), and media with dimethyl sulfoxide (DMSO) served as positive control (-▪-) (B). Mean ± SEM. * p<0.05. [file Image_3.jpeg]

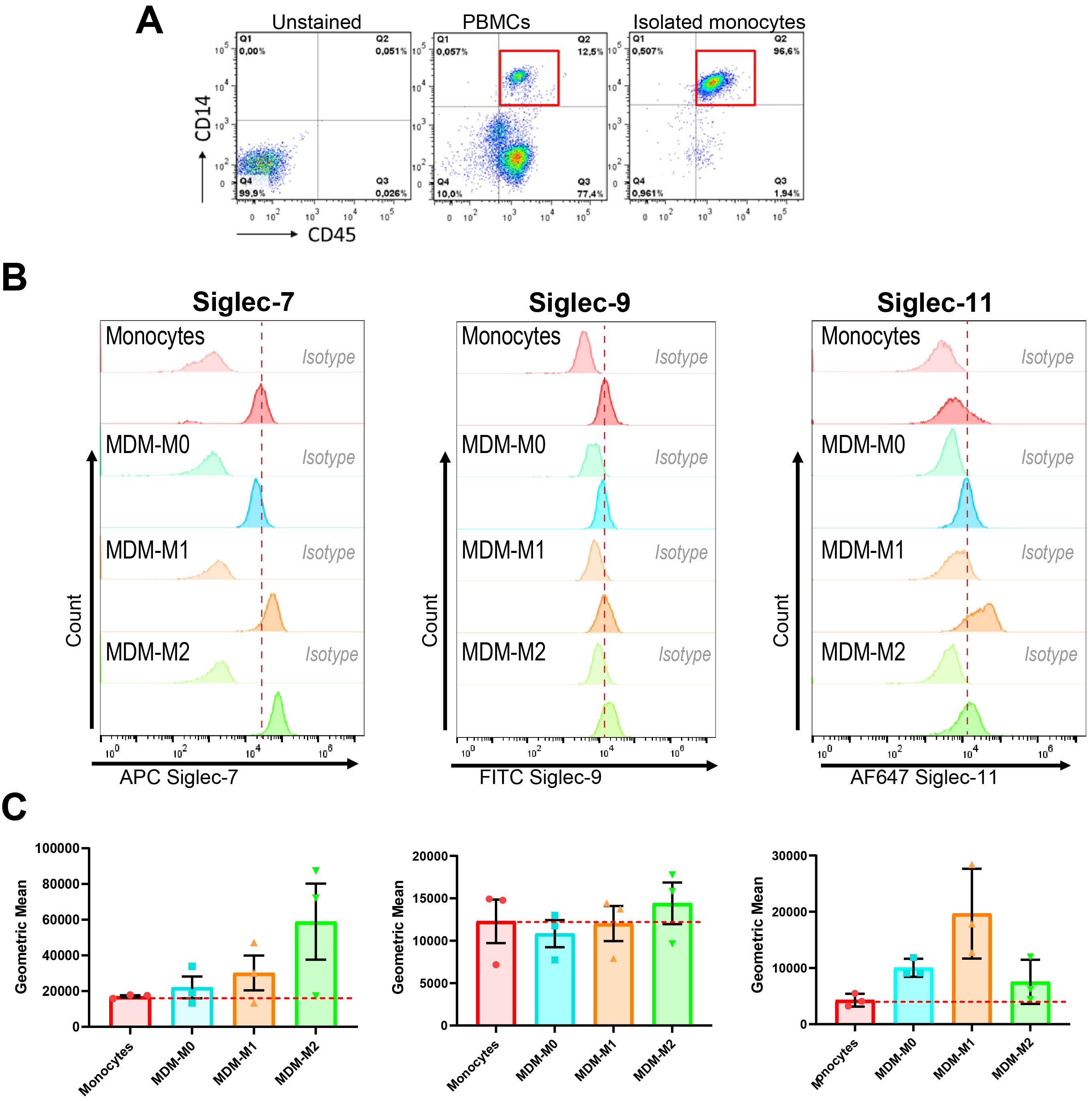

Supplement: Supplementary Figure 4 — Siglec expression on monocyte derived macrophages (MDM). Monocytes were isolated from human PBMCs were stained for CD14 and CD45 to determine the purity (A). The expression of Siglec-7, -9 and -11 was determined by flow cytometry on M0-MDM, M1-MDM and M2-MDM and compared to monocytes (red dash line) from 3 different PBMCs donors. Representative histograms for Siglec-7, -9 and -11 intensity on M0-MDM, M1-MDM and M2-MDM Mare showing (top) and geometric means from 3 normal donors (bottom) (B, C). Mean ± SEM. [file Image_4.jpeg]

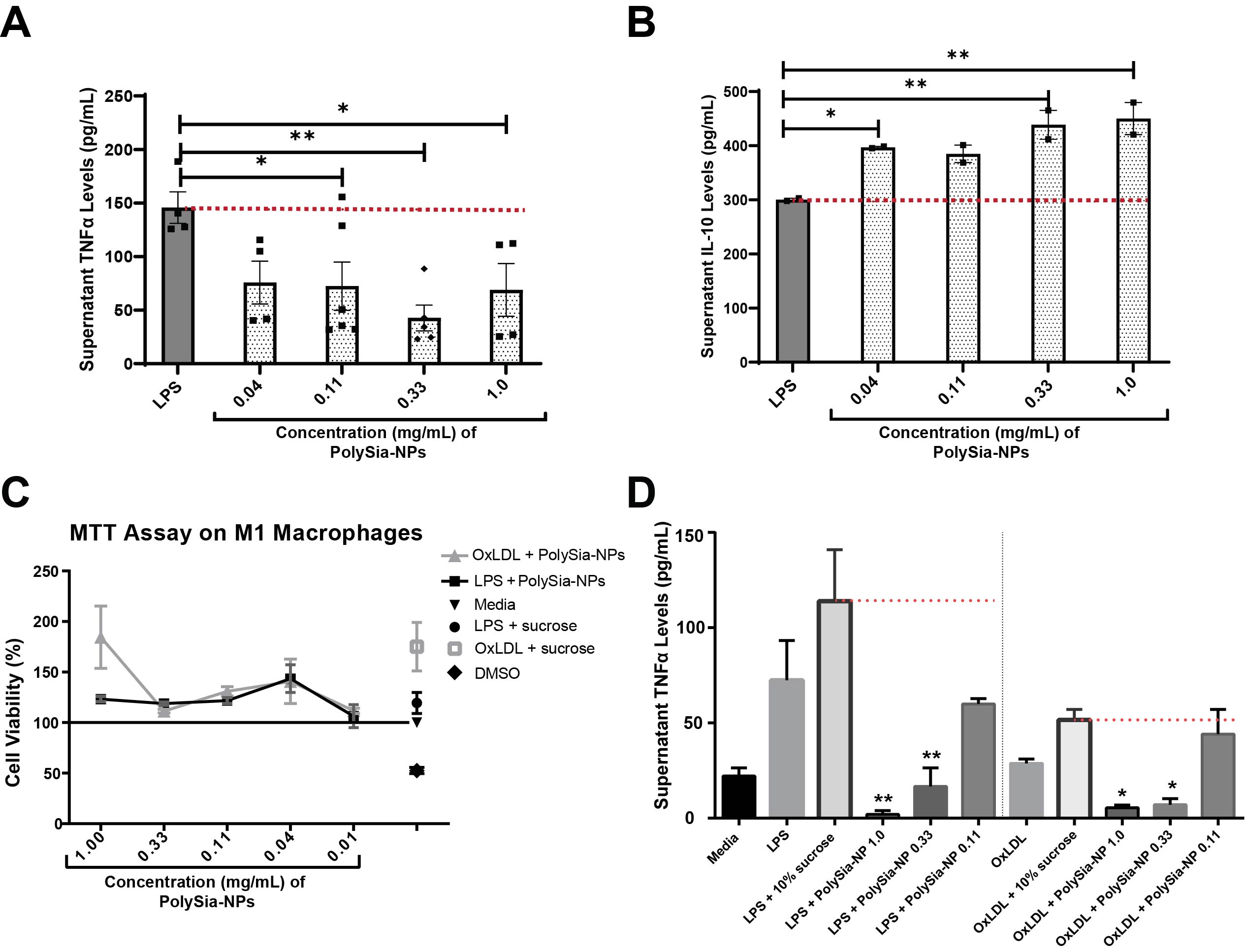

Supplement: Supplementary Figure 5 — Cytokine measurement from human THP-1-derived macrophages supernatant. TNF-α (A) and IL-10 (B) protein concentrations were determined by ELISA assay from THP-1 macrophages supernatant treated with serial dose range of PolySia-NPs (0.04-1.0 mg/mL) in presence of LPS overnight. LPS served as a positive control. Viability assessments on LPS vs OxLDL stimulated M1-macrophages determined by the colorimetric MTT assay. M1-MDM macrophages were treated with either LPS or OxLDL and serial dose range of PolySia-NPs (1.0-0.11mg/mL). Results are shown as % viability where media alone served as internal negative control (-♦-) (100% viable), and media with dimethyl sulfoxide (DMSO) served as positive control (-♦-) (C). TNF-α was determine by ELISA on cell supernatant form M1-MDM macrophages treated with either LPS or OxLDL and serial dose range of PolySia-NPs (D). Mean ± SEM. * p<0.05, **p<0.01. [file Image_5.jpeg]

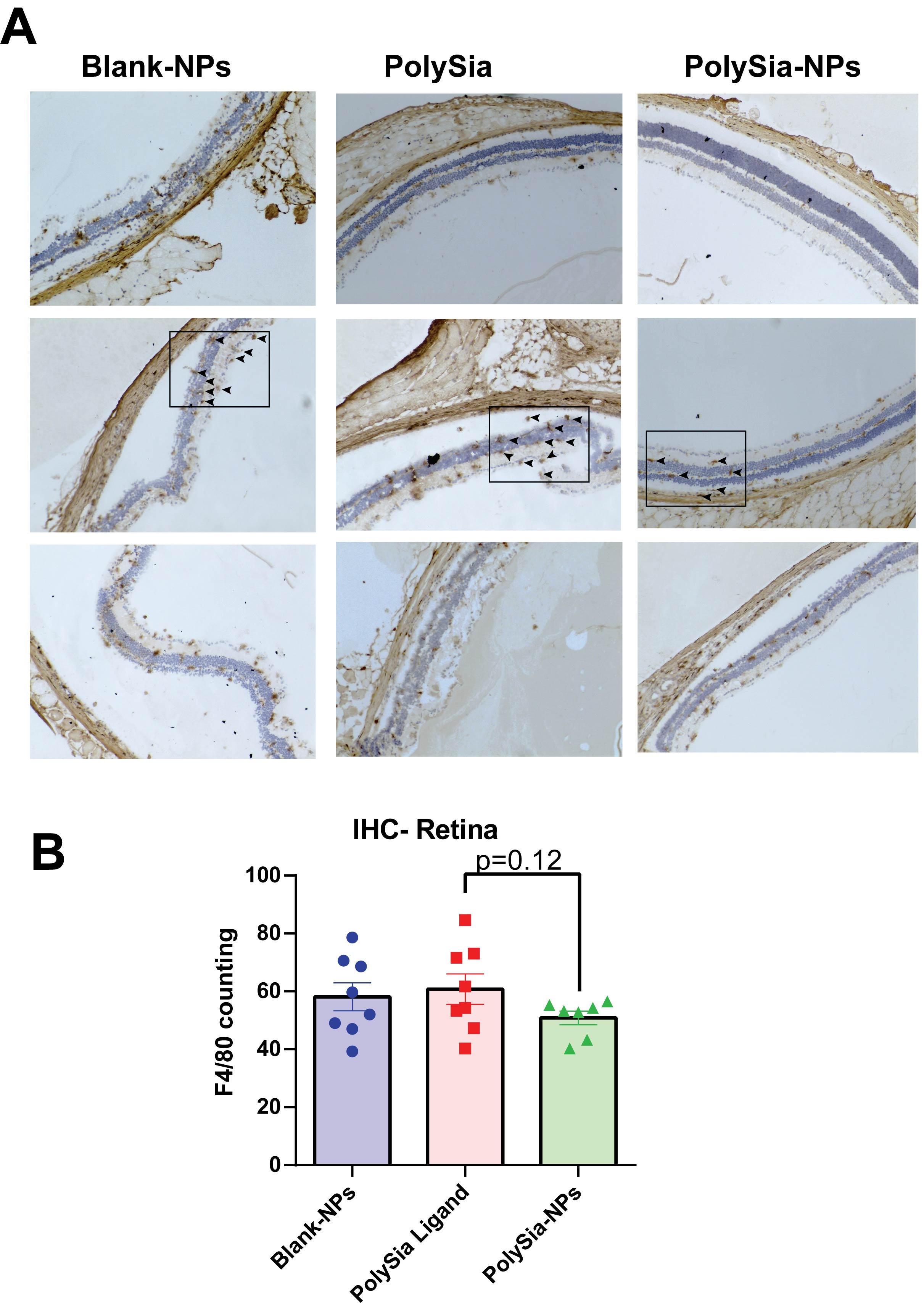

Supplement: Supplementary Figure 6 — Immunohistochemistry (IHC) for F4/80 staining in retinas from BLD model. Three representative figures of retinas stained for F/80 macrophages from Blank-NP, PolySia ligand and PolySia-NPs treated groups in BLD mice. Black arrows show F/480+ macrophages in a representative square area (A). Quantification of F/80 staining from Blank-NP (blue), PolySia ligand (red) and PolySia-NPs (green) treated retinas from BLD mice (B). NPs: nanoparticles. [file Image_6.jpeg]

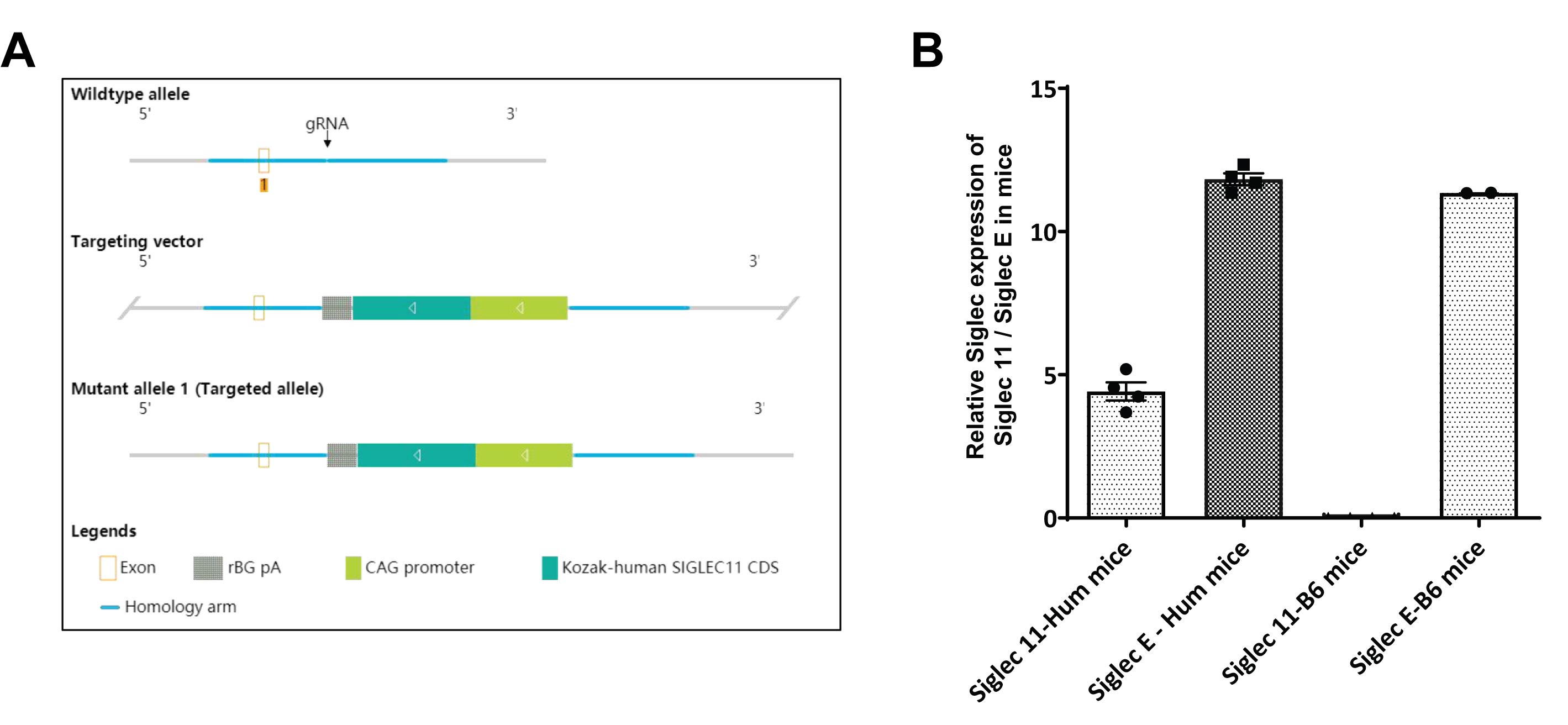

Supplement: Supplementary Figure 7 — Human Siglec-11 transgenic mice production. Overview of the Targeting Strategy showing the CAG promoter-Kozak-human SIGLEC11 CDS-rBG pA” cassette cloned into intron 1 of ROSA26 in a C57BL/6N mice by CRISPR/Cas-mediated genome engineering (A). RT-PCR analysis showing the expression of human Siglec-11 and mouse Siglec-E in the transgenic hSiglec-11 mice and wild type C57BL/6N mice (B). [file Image_7.jpeg]

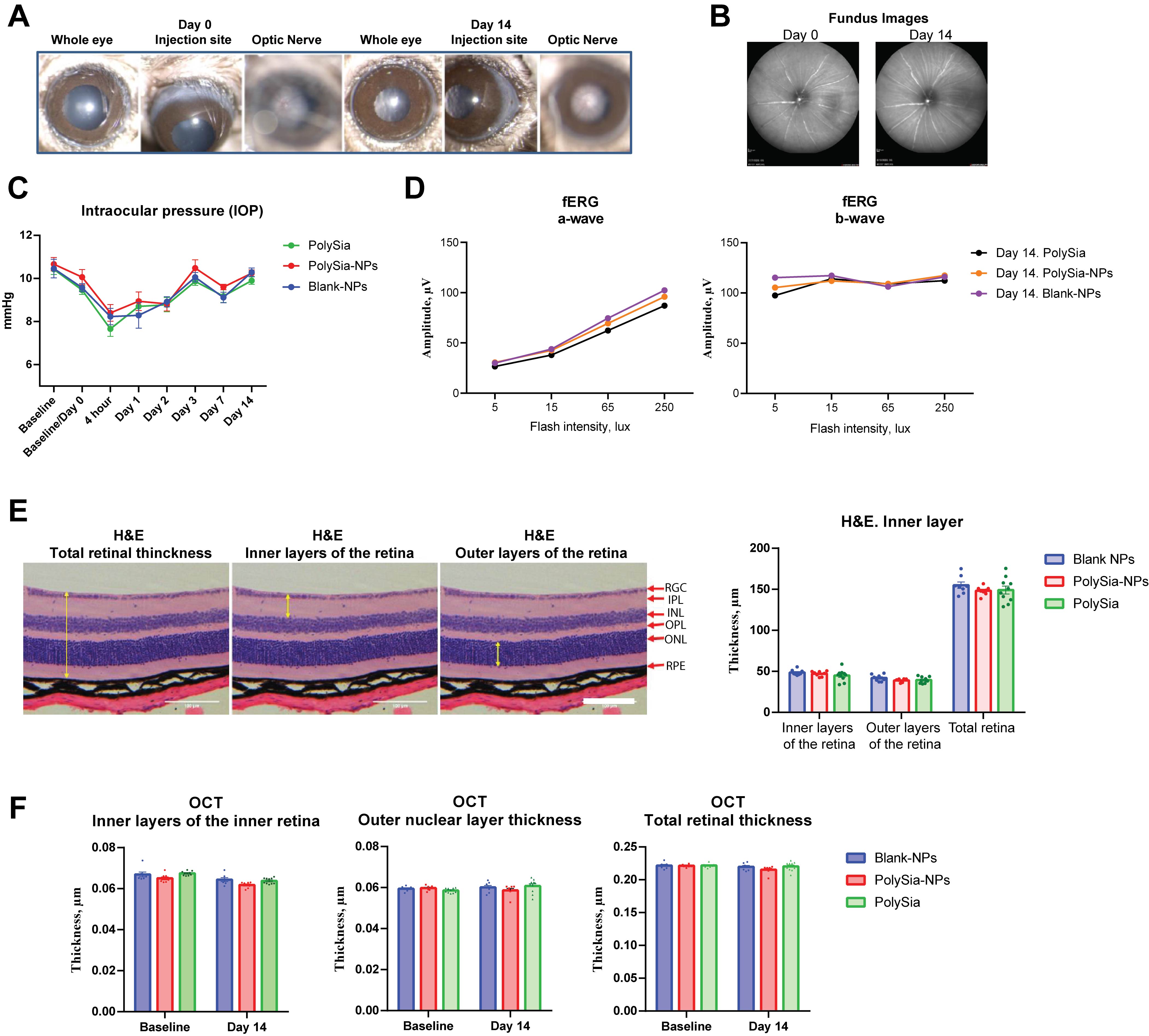

Supplement: Supplementary Figure 8 — In vivo clinical and histological evaluations after intravitreal (IVT) injections in mice. Slit lamp images of whole eye, injection site at day 0 and 14, and optic nerve (A), fundus images at day 0 and 14 (B), Intraocular presure measurments at baseline, day 0 (baseline), 4h and day 1, 2, 3, 7 a 14 after injection with Blank-NPs (blue), PolySia-NPs (red) and sialic acid ligand (green) (C). fERG a-wave and b-wave and after exposing to flash of light at 2, 15, 65 and 250 lux of intensity after injection with blank NPs (blue and purple), PolySia-NPs (red and orange) and PolySia ligand (green, black) at day 0 (baseline) and 14 respectively (D). Histological sections (H&E) showing the retinal layers and thickness quantification of inner nuclear layer, outer layers and total retinal, (E) of the retina. OCT thicknesses at day 0 (baseline) and 14 after injection with blank-NPs (blue), PolySia-NPs (red) and PolySia ligand (green), measured from SD-OCT scans (F). Analysis made by Two-way ANOVA, p>0.05 for all. RGC: Retinal Ganglion Cell Layer, IPL: Inner Plexiform Layer, INL: Inner Nuclear Layer, ONL: Outer Nuclear Layer, RPE: Retinal Pigmented Epithelium/Bruch’s Membrane Choroid. White bar: 100μm. Data is presented as mean ± SEM. NPs, nanoparticles. [file Image_8.jpeg]

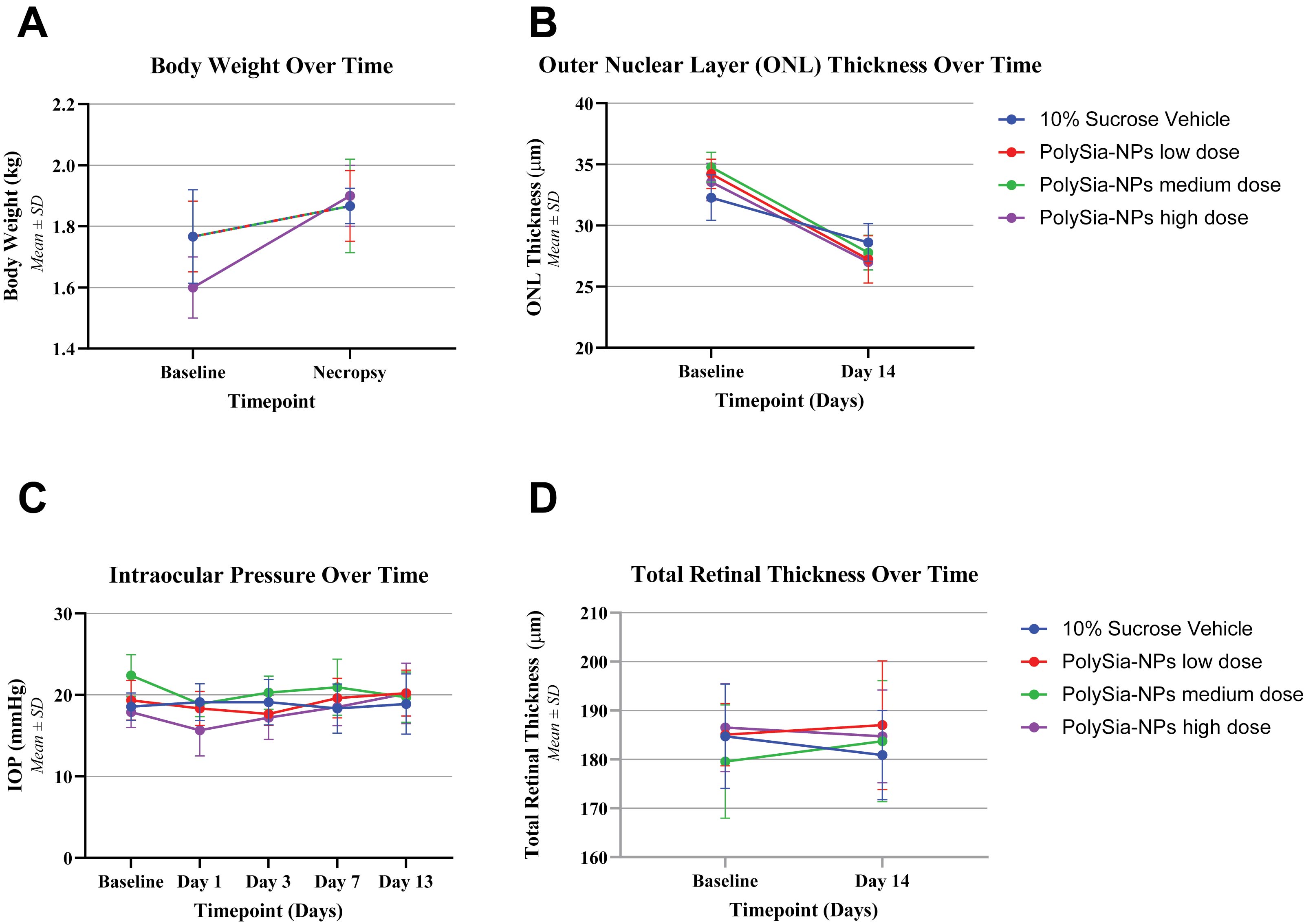

Supplement: Supplementary Figure 9 — Clinical evaluations after intravitreal (IVT) inoculations in rabbits at baseline and at day 14 after injection with vehicle (blue) or PolySia-NPs at low (red), medium (green) and high (magenta) dose. Change in Body Weight Over Time (A). Optical Coherence Tomography (OCT) imaging of the posterior section of the eye. Change in Outer Nuclear Layer Thickness (B) Intraocular Pressure (C) and Total Retina thickness over the time (D). NPs, nanoparticles. [file Image_9.jpeg]
